# A Core‐Brush Nanoplatform with Enhanced Lubrication and Anti‐Inflammatory Properties for Osteoarthritis Treatment

**DOI:** 10.1002/advs.202406027

**Published:** 2024-11-01

**Authors:** Yingying Liu, Zhiyan Ma, Xin Wang, Jiaming Liang, Linlin Zhao, Yingyu Zhang, Jiayu Ren, Shuping Zhang, Yajun Liu

**Affiliations:** ^1^ Biomedical Sciences College Shandong Medicinal Biotechnology Centre Shandong First Medical University & Shandong Academy of Medical Sciences Jinan Shandong 250117 China; ^2^ Medical Science and Technology Innovation Center Shandong First Medical University & Shandong Academy of Medical Sciences Jinan Shandong 250117 China; ^3^ Faculty of Light Industry, State Key Laboratory of Biobased Material and Green Papermaking Qilu University of Technology (Shandong Academy of Sciences) Jinan 250353 China; ^4^ School of Public Health Shandong First Medical University & Shandong Academy of Medical Sciences Jinan Shandong 250117 China; ^5^ National Center for Orthopaedics Beijing Research Institute of Traumatology and Orthopedics Beijing Jishuitan Hospital Capital Medical University Beijing 100035 China

**Keywords:** controlled release, drug delivery, enhanced lubrication, inflammatory regulation, osteoarthritis

## Abstract

Osteoarthritis (OA) is recognized as a highly friction‐related joint disease primarily associated with increased joint friction and inflammation due to pro‐inflammatory M1‐type macrophage infiltration in the articular cavity. Therefore, strategies to simultaneously increase lubrication and relieve inflammation to remodel the damaged articular microenvironment are of great significance for enhancing its treatment. Herein, a multifunctional core‐brush nanoplatform composed of a ROS‐scavenging polydopamine‐coated SiO_2_ core and lubrication‐enhancing zwitterionic poly(2‐methacryloyloxyethyl phosphorylcholine) (PMPC) brush and loaded with the anti‐inflammatory drug curcumin by a reactive oxygen species (ROS)‐liable conjugation (named as SiO_2_@PP‐Cur) is rationally designed. Benefiting from the grafted zwitterionic PMPC brush, a tenacious hydration layer with enhanced lubricity for reducing joint abrasions is developed. More importantly, based on the mono‐iodoacetic acid‐induced arthritis (MIA) rat model, intra‐articular injection of SiO_2_@PP‐Cur nanoplatform can effectively alleviate articular inflammation via promoting macrophage polarization from the pro‐inflammatory M1 to anti‐inflammatory M2 state by activating the nuclear factor erythroid 2‐related factor 2 (Nrf2) signaling pathway and attenuating the degradation of cartilage matrix, resulting in the remodeling of the damaged microenvironment into a pro‐regenerative microenvironment. As a result, SiO_2_@PP‐Cur can considerably inhibit OA progression. Therefore, the work may provide a novel strategy for the development of an advanced core‐brush nanoplatform for enhanced OA therapy.

## Graphical abstract

A core‐brush nanoplatform containing microenvironment‐triggered curcumin conjugates, reactive oxygen species (ROS)‐scavenging polydopamine coating, and lubrication‐enhanced zwitterionic brushes are fabricated for the synergistic treatment of osteoarthritis. With their combined features, the nanoplatform can effectively alleviate cartilage wear, limit inflammatory reactions, and reduce articular cartilage breakdown and the degradation of extracellular matrix during the progression of osteoarthritis by concomitantly exerting anti‐inflammatory and lubricating effects.

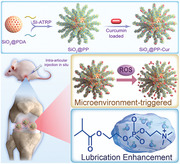

## 1 Introduction

Osteoarthritis (OA) is a highly friction‐related joint disease induced by the breakdown of articular cartilage and inflammation of the joint capsule and is characterized by chondrocyte apoptosis, cartilage matrix degradation, and subchondral bone damage.^[^ ^1^, ^2^, ^3^ ^]^ The clinical therapies include oral or intravenous injections of anti‐inflammatory drugs or intra‐articular injections of hyaluronic acid (HA) that mitigates articular surface friction.^[^ ^4^, ^5^ ^]^ Nonetheless, frequent usage of anti‐inflammatory drugs may result in side effects, such as gastrointestinal reactions and liver injury.^[^ ^6^ ^]^ Hyaluronic acid's shear‐thinning characteristic with regard to the elevated joint motion shear rate, as well as its degradation in the presence of hyaluronidase, may also lead to compromised lubrication.^[^ ^7^ ^]^ Meanwhile, surgical treatment at the later stage of OA causes physical trauma and may also pose a heavy economic burden for the patient.^[^ ^8^ ^]^ Recently, nanotherapy approaches with synergistic lubrication and anti‐inflammatory effects have shown promise in effectively alleviating cartilage wear, suppressing inflammation, and enhancing treatment outcomes for OA.^[^ ^9^, ^10^ ^]^ However, the rational design of these systems remains challenging.^[^ ^11^ ^]^ Firstly, the optimal cartilage lubricant should be a multifunctional composite material that integrates mechanical support and lubrication properties in order to mimic the low‐friction characteristics and high load‐bearing capacity of natural cartilage.^[^ ^12^, ^13^ ^]^ Furthermore, the uncontrolled release of therapeutic drugs in vivo often poses risks of adverse side effects. Therefore, the development of a multifunctional nanoplatform with excellent mechanical load‐bearing and joint lubrication properties, endogenous response for on‐demand release of loaded drugs, and the regulation of joints inflammatory microenvironment is of significant importance for enhancing treatment efficacy in early OA.

Natural articular cartilage is a robust and elastic tissue characterized by exceptional low‐friction properties and high load‐bearing capacity. The high stiffness and toughness of natural cartilage from an entangled network of collagen fibers and proteoglycans, serving an essential role akin to that of a “shock absorber”.^[^ ^12^, ^13^ ^]^ Consequently, the optimal cartilage lubricant should be a multifunctional composite material that integrates mechanical support and lubricative properties. Primarily, such a lubricant ought to be a “soft” material, facilitating the formation of a hydrophilic lubrication layer that effectively minimizes interfacial friction. Furthermore, cartilage lubricating materials should also possess “hard” properties, including load‐carrying capacity and extreme‐pressure resistance, to sustain wear resistance under conditions of elevated stress.^[^ ^14^, ^15^ ^]^ The low friction of natural cartilage has been attributed to the phosphatidylcholine (PC) lipids of nonfluid boundary layers, whose highly hydrated phosphocholine headgroups may reduce friction through the hydration lubrication mechanism.^[^ ^16^ ^]^ Inspired by the natural biological lubricant, the lubrication performance of biomimetic brush polymers has been widely studied and applied to achieve an excellent superlubricity in articular cartilage.^[^ ^17^ ^]^ Particularly, compared to linear polymer chains with equivalent molar mass, brushes have higher grafting density and robust polymer brushes, thereby achieving better lubrication performance. This might be due to the more uniform brush polymer and the higher hydration level in the grafted layer of the brush on the substrate. Nanoplatforms coated by zwitterionic brush polymer have hydration layers on their outer surfaces and can provide efficient lubrication. Mechanically, the water molecules within the hydration layers are tenaciously held due to the interaction of the large water dipole with the enclosed charge, leading to a response in a fluid‐like manner when being sheared.^[^ ^5^ ^]^ Therefore, based on the hydration effects of zwitterionic polymer brushes and the rigidity of silica nanoparticles, we modified zwitterionic polymer brush layers to the silica periphery. The “soft” zwitterionic polymer brush layers can enhance the lubrication performance of the material through hydration lubrication, while the “hard” silica nanoparticles are capable of bearing high shear forces under joint motion, contributing to the anti‐wear resistance.

Inflammatory responses of OA are typically related to oxidative stress and pro‐inflammatory cytokines secretion in the OA microenvironment.^[^ ^18^, ^19^ ^]^ A growing body of evidence has demonstrated that macrophages play a vital role in the symptomology and structural progression of OA.^[^ ^20^, ^21^ ^]^ In response to microenvironmental stimulation, the two major phenotypes of macrophages are pro‐inflammatory M1‐type and anti‐inflammatory M2‐type.^[^ ^22^, ^23^ ^]^ More specifically, M1 macrophages predominantly promote inflammation and articular degeneration through the secretion of pro‐inflammatory cytokines (including tumor necrosis factor α (TNF‐α), interleukin‐1β (IL‐1β) and interleukin‐6 (IL‐6)).^[^ ^24^ ^]^ On the other hand, M2 macrophages secrete anti‐inflammatory cytokines (e.g., interleukin‐10 (IL‐10), interleukin‐4 (IL‐4), and transforming growth factor‐β (TGF‐β)), thereby inhibiting the function of M1 macrophages.^[^ ^25^ ^]^ Accordingly, weakening the microenvironment's proinflammatory response and inducing macrophage shift from the M1 to the M2 state is a potentially viable approach for the treatment of OA. Curcumin, the active component of Curcuma longa, can significantly inhibit cell apoptosis induced by oxidative stress in OA and other disorders owing to its remarkable anti‐oxidative and anti‐inflammatory activities.^[^ ^26^, ^27^, ^28^ ^]^ However, curcumin with a small molecular weight is easily removed by capillaries and lymphatic vessels.^[^ ^19^, ^29^, ^30^ ^]^ Moreover, the uncontrolled release of therapeutic drugs in vivo always carries the risk of untoward side effects.^[^ ^31^, ^32^ ^]^ Accumulating evidence demonstrated that reactive oxygen species (ROS) accumulate in the arthritic microenvironment and promote the development of osteoarthritis.^[^ ^33^ ^]^ Therefore, we expected that ROS‐responsive curcumin conjugates could be used as controllable release therapeutic agents in the articular environment.

**Scheme 1:**
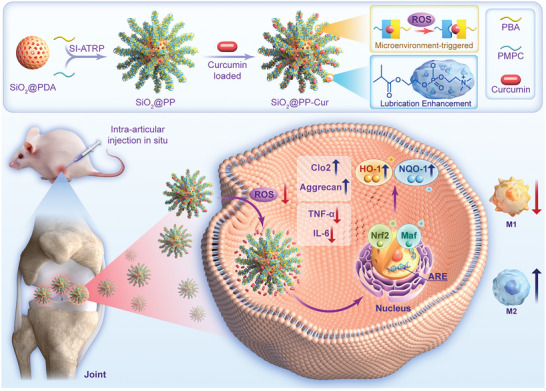
The schematic diagram for the design and mechanistic routes of SiO_2_@PP‐Cur NPs for the treatment of OA.

In the current study, a core‐brush nanoplatform, namely SiO_2_@PP‐Cur, that contained microenvironment‐triggered curcumin conjugates, ROS‐scavenging polydopamine coating, and lubrication‐enhanced zwitterionic brushes was developed for the synergistic treatment of osteoarthritis (**Scheme** **1**). First, owing to the microenvironment‐triggered cleavage of dynamic linkage among the curcumin 1,3‐diketone group and phenylboronic acid (PBA) moieties, the core‐brush nanoplatform containing curcumin conjugates would be gradually degraded to release the loaded curcumin, which possesses anti‐inflammatory and anti‐oxidative properties. Second, the “soft” polyzwitterionic branches of poly(2‐methacryloyloxyethyl phosphorylcholine) (PMPC) could endow the nanoplatforms with enhanced lubricity by forming a tenacious hydration layer around the PO_4_^−^ and N^+^(CH_3_)_3_ groups in PMPC brushes. While the “hard” silica nanoparticles are capable of bearing high shear forces under joint motion, contributing to the anti‐wear resistance. Third, the prepared core‐brush nanoplatform could remarkably shift M1 macrophage polarization to the anti‐inflammatory M2 phenotype, hence switching the impaired microenvironment to a pro‐regenerative microenvironment. As a result, the therapeutic results based on the rat model of OA indicated that intra‐articular injection of SiO_2_@PP‐Cur exhibited a promising synergistic therapeutic effect.

## 2 Results and Discussion

### 2.1 Characterization of SiO_2_@PP‐Cur

**Figure 1:**
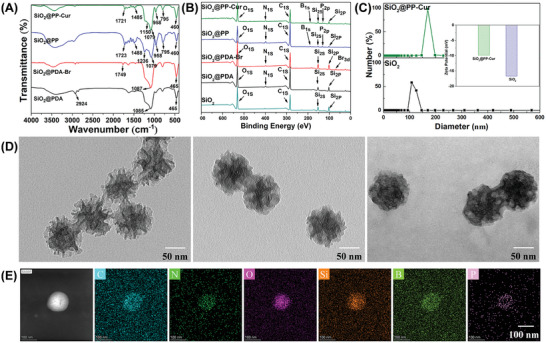
Characterization of SiO_2_@PP‐Cur NPs. A,B) The FT‐IR and XPS spectra of SiO_2_@PDA, SiO_2_@PDA‐Br, SiO_2_@PP, and SiO_2_@PP‐Cur, respectively. C) Hydrodynamic diameters and the zeta‐potential of SiO_2_ and SiO_2_@PP‐Cur. D) Representative TEM images of SiO_2_, SiO_2_@PDA, and SiO_2_@PP‐Cur. E) EDS elemental mapping for SiO_2_@PP‐Cur.

SiO_2_@PP‐Cur NPs were prepared through dopamine‐mediated surface‐initiated atom transfer radical polymerization (SI‐ATRP) and then coupled with curcumin (Figure S1, Supporting Information). According to **Figure** **1**A, Fourier transform infrared (FT‐IR) spectroscopy features a peak at 1091 cm^−1^ of the SiO_2_ NPs that is attributable to Si‐O‐Si absorption.^[^ ^34^ ^]^ For SiO_2_@PDA‐Br, the adsorption band at 1749 cm^−1^ represented the amide condensation reaction between SiO_2_@PDA and 2‐bromoisobutyryl bromide (BiBB). In addition, compared with the pristine SiO_2_ NPs, the spectrum of SiO_2_@PP features four peaks of P═O stretching (1236 cm^−1^), P‐O shearing (958 cm^−1^), C═O stretching (1723 cm^−1^), and B‐O stretching (1488 cm^−1^), implying the effective growth of PMPC and PBA brushes.^[^ ^35^, ^36^ ^]^ Besides, the spectrum of SiO_2_@PP‐Cur NPs features a peak at 1150 cm^−1^ that coincides with the C‐O stretching vibration, suggesting the successful conjugation of PBA and curcumin.^[^ ^37^ ^]^ The X‐ray photoelectron spectroscopy (XPS) spectrum of SiO_2_ illustrated in Figure 1B features peaks at 153 and 101 eV that represent the orbitals of Si 2s and Si 2p, respectively.^[^ ^38^ ^]^ Compared with the SiO_2_@PDA, the survey spectrum of SiO_2_@PDA‐Br showed the existence of Br 3d peak (at ≈70 eV), illustrating the immobilization of bromide initiator. Furthermore, two peaks of B 1s (at ≈189 eV) and P 2p (132 eV) appeared in the spectrum of SiO_2_@PP‐Cur, demonstrating the existence of PMPC and PBA brush structures.^[^ ^36^, ^39^ ^]^ Furthermore, thermogravimetric analyses (TGA) were performed to evaluate the PMPC‐PBA brushes grafting ratio (Figure S3, Supporting Information). Compared with SiO_2_@PDA, the overall percentage of weight loss for SiO_2_@PP was increased by 40.72%, which corresponded to the thermal degradation of PMPC‐PBA brushes. The SI‐ATRP on the SiO_2_ was also evaluated through the free PMPC‐PBA co‐polymer brushes generated by sacrificial coinitiator. As displayed in Figure S4 (Supporting Information), the peaks at 7.0‐8.0 ppm are the characteristic signals of the benzene ring in PBA moiety. Additionally, the peak at 2.9 ppm belongs to the quaternary ammonium group in PMPC moiety. According to the ^1^H NMR spectrum of PMPC‐PBA brushes, the grafting ratios of PMPC brushes and PBA brushes were 28.19% and 12.53%, respectively. Transmission electron microscopy (TEM) depicted that SiO_2_@PP‐Cur NPs manifested a spherical, shell‐core morphology (Figure 1D). At the same time, elemental mapping demonstrated the uniform decoration of PBA and PMPC brushes (Figure 1E). The hydrodynamic size of SiO_2_@PP‐Cur was concentrated at 140 nm, and the ζ‐potential was −10.40 mV (Figure 1C), while the drug loading content was 6.58 ± 1.03%, as calculated by ^1^H NMR and HPLC (Figures S4 and S5, Supporting Information). Moreover, the SiO_2_@PP‐Cur NPs displayed excellent stability in PBS for 14 days at 37 °C (Figure S6, Supporting Information).

### 2.2 Evaluation of Lubrication Performance, Drug Release, and ROS Scavenging in Vitro

**Figure 2:**
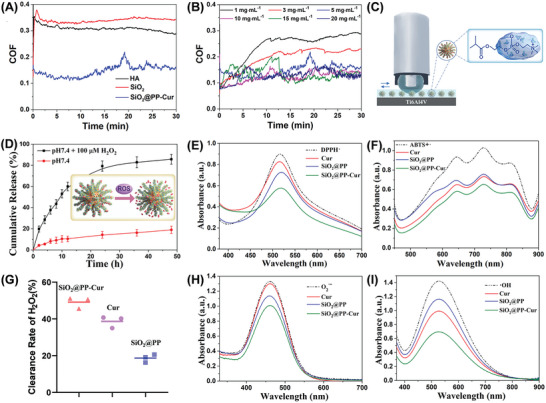
In vitro assays of lubricity, drug release, and ROS scavenging properties. A) Friction coefficient‐time plots of HA, SiO_2_, and SiO_2_@PP‐Cur. B) Lubrication properties of different concentrations of SiO_2_@PP‐Cur. C) Schematic illustration of the lubricating performance of SiO_2_@PP‐Cur. D) Release profiles of SiO_2_@PP‐Cur NPs in PBS in the presence or absence of 100 µm H_2_O_2_ at 37 °C. G) The influence of different nano‐formulations on the scavenging effect of H_2_O_2_ under identical conditions. UV–vis absorbance spectra of E) DPPH·, F) ABTS+·, H) O_2_^·−^, I) ·OH radicals after incubation with different nano‐formulations under identical conditions.

Furthermore, the ability of SiO_2_@PP‐Cur to enhance lubrication was tested by investigating its friction coefficient. Interestingly, the results demonstrated that the friction coefficient of SiO_2_@PP‐Cur was 0.15 (loading force: 5 N; reciprocating frequency: 3 Hz; concentration: 5 mg·mL^−1^), lower than that of SiO_2_ (0.35) and hyaluronic acid (0.32), implying its superior lubricity (**Figure** **2**A). We also monitored the friction coefficient of SiO_2_@PP‐Cur at different concentrations, loading forces, and reciprocating frequency. Figure 2B shows that the friction coefficient of SiO_2_@PP‐Cur slightly decreases with increasing concentration. In addition, the lubrication performance of SiO_2_@PP‐Cur under different loading forces (3 Hz, 5 mg·mL^−1^) and reciprocating frequency (5 N, 5 mg·mL^−1^) was also examined (Figure S7, Supporting Information). There is no significant change of the friction coefficient with the increase of loading forces and reciprocating frequency, indicating that the lubrication performance of nanoplatform is very stable. Inspired by the superior load‐bearing capabilities and the intrinsic surface lubrication mechanism of articular cartilage, we grafted a “soft” lubrication‐enhancing zwitterionic polymer brush layer onto the surface of a “hard” silica nanoparticle. This engineered surface zwitterionic brush layer contributes to effective hydration‐based lubrication, while the SiO_2_ serves as a robust substrate, offering significant load‐bearing support. Based on the hydration lubrication theory, PMPC branches with a polyzwitterionic structure and endows the nanoplatform with enhanced lubricating properties by forming a tenacious hydration layer around the PO_4_^−^ groups and N^+^(CH_3_)_3_ in PMPC, thereby leading to a significant reduction of friction coefficient value.^[^ ^16^, ^17^ ^]^ The SiO_2_ spherical nanoparticle exhibits the propensity to function as rolling elements when encountering local asperities, ultimately resulting in the formation of a boundary film. This boundary film is capable of providing support for load and effectively reducing the interfacial friction. Therefore, upon the administration of SiO_2_@PP‐Cur in the space within the joint cavity, the hydrated layer that is generated by PMPC during joint movement is able to withstand the significant shear force and enables SiO_2_@PP‐Cur to exert a lubricating effect owing to hydration.

For the purpose of examining the controllable release of SiO_2_@PP‐Cur, the conditioned release efficiency of curcumin was analyzed in vitro. As displayed in Figure 2D, the release of curcumin from SiO_2_@PP‐Cur was 78.20% following the administration of 100 µm H_2_O_2_ for 48 h. In parallel, only ≈23.52% of curcumin was secreted at a physiologic pH of 7.4, demonstrating the ROS‐responsive release of curcumin from SiO_2_@PP‐Cur. These results revealed that SiO_2_@PP‐Cur was beneficial in improving the utilization of curcumin.

As is well documented, curcumin is a natural reducing agent extracted from plants, and polydopamine is rich in reductive groups such as catechol and quinone moieties, and thus we speculated that the synthesized SiO_2_@PP‐Cur might exhibit a high ROS‐scavenging effect. Next, H_2_O_2_, 1,1‐diphenyl‐2‐picrylhydrazyl (DPPH·), 2,2′‐azinobis (3‐ethylbenzthiazoline‐6‐sulfonate) radicals (ABTS+·), hydroxyl radical (·OH), and superoxide anion (O_2_^·−^) were employed to explore the anti‐oxidative efficiency of SiO_2_@PP‐Cur. The analysis determined that the radicals were efficiently removed by SiO_2_@PP‐Cur (Figure 2E–I). Attributed to the abundant reductive groups of polydopamine coating and the anti‐oxidative activity of curcumin, SiO_2_@PP‐Cur displayed substantial ROS‐scavenging ability. Furthermore, SiO_2_@PP‐Cur still exhibited outstanding antioxidative capability after 6 months (Figure S8, Supporting Information). Collectively, these observations insinuated that SiO_2_@PP‐Cur could serve as a potent ROS‐scavenging agent against oxidation.

### 2.3 Cytotoxic, Anti‐Inflammatory, and Antioxidative Properties of SiO_2_@PP‐Cur at the Cellular Level

**Figure 3:**
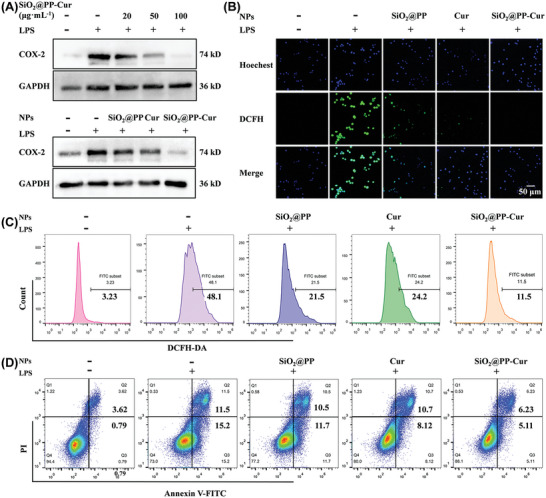
Assays on the anti‐inflammatory and anti‐oxidative efficacy of SiO_2_@PP‐Cur NPs. A) Protein expression levels of COX‐2 based on Western blot analysis of RAW264.7 cells supplemented with NPs for 24 h (SiO_2_@PP‐Cur: 50 µg·mL^−1^, *n* = 3). B) Confocal laser scanning microscope (CLSM) images of macrophages receiving different treatments for 24 h (green: DCFH; blue: nuclei) (*n* = 3). Flow cytometry analysis of C) ROS levels in macrophages 24 h post‐incubation with various reagents (*n* = 3), and D) the number of apoptotic cells in macrophages incubated for 24 h with various reagents (*n* = 3).

As established by the cytotoxicity assay, SiO_2_@PP‐Cur elicited no significant cytotoxicity in macrophages (Raw264.7 was selected) even at a higher concentration (≈400 µg·mL^−1^) for 24 h, indicating the favorable biocompatibility of these core‐brush nanoplatforms (Figure S9, Supporting Information). Cyclooxygenase‐2 (COX‐2) is highly involved in the inflammatory response.^[^ ^19^, ^40^ ^]^ To begin, macrophages were stimulated with lipopolysaccharide (LPS), a strong activator that is usually applied to immune cells.^[^ ^41^, ^42^ ^]^ As depicted in **Figure** **3**A, SiO_2_@PP‐Cur treatment significantly down‐regulated LPS‐stimulated COX‐2 expression in a dose‐dependent manner in macrophages. SiO_2_@PP‐Cur elicited superior efficiency in inhibiting inflammatory response compared to SiO_2_@PP and curcumin (Cur). Afterward, the anti‐oxidative and anti‐apoptotic effects of SiO_2_@PP‐Cur were explored. As opposed to the control group, the proportion of macrophages with excessive levels of ROS production was significantly increased after LPS stimulation for 24 h (Figures S10 and S11, Supporting Information). Besides, SiO_2_@PP‐Cur decreased ROS levels in LPS‐treated macrophages in a dose‐dependent fashion, with an effective reduction in ROS level at 50 mg•mL^−1^. Furthermore, SiO_2_@PP‐Cur exhibited higher efficiency in scavenging ROS than SiO_2_@PP and Cur (Figure 3B,C). What's more, SiO_2_@PP‐Cur significantly reduced the proportion of early and late apoptotic macrophages induced by LPS treatment, while SiO_2_@PP and Cur could only partially decrease the proportion of apoptotic cells (Figure 3D; Figure S13, Supporting Information). Taken together, these results validated the promising anti‐oxidative and anti‐apoptotic effects of SiO_2_@PP‐Cur on macrophages. In summary, SiO_2_@PP‐Cur exhibited low cytotoxicity and superior efficacy in anti‐inflammatory, anti‐apoptotic, and ROS‐scavenging effects. Considering that the lubricating effect of PMPC could not be examined in vitro, SiO_2_@PP‐Cur principally utilizes polydopamine coating and releases curcumin to inhibit macrophage activation and mediate oxidative stress in order to ameliorate osteoarthritis‐induced inflammation.

### 2.4 Microenvironment Regulatory Capacity of SiO_2_@PP‐Cur In Vitro

**Figure 4:**
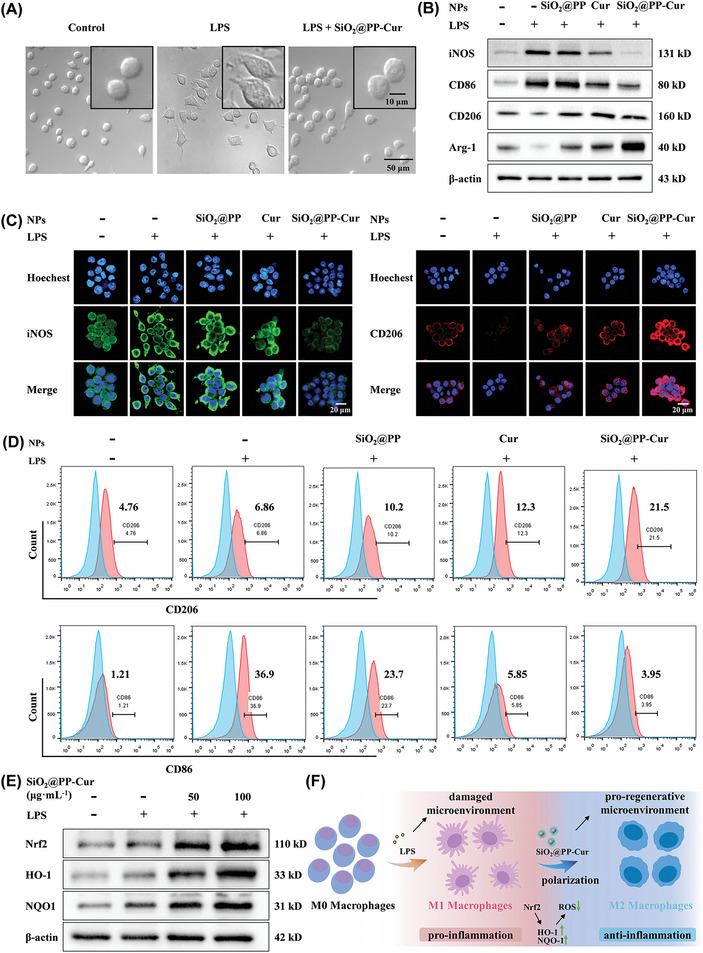
Macrophage phenotype reprogramming. A) Optical microscopic images comparing morphological characteristics of macrophages and LPS‐induced macrophages with or without SiO_2_@PP‐Cur treatment for 48 h. B) Relative protein expression of M1‐related markers and M2‐specific markers in M1‐polarized macrophages following incubation with different groups for 24 h (*n* = 3). C) Immunofluorescence staining images of LPS‐induced macrophages incubated for 24 h with various treatments (red: CD206; green: iNOS; blue: nuclei) (*n* = 3). D) Representative flow cytometry results of CD86 and CD206 expression levels in macrophages incubated for 24 h with various reagents (*n* = 3). E) Protein expression levels of Nrf2, HO‐1, and NQO‐1 in macrophages as identified by western blotting (*n* = 3). F) The possible anti‐inflammatory mechanism and macrophage phenotypic reprogramming.

Given that macrophages, particularly M1 macrophages, participate in OA progression, the impact of SiO_2_@PP‐Cur on the inflammatory response by altering macrophage polarization (Raw264.7 was selected) was determined. As delineated in **Figure** **4**A, LPS‐treated macrophages exhibited a multiple tentacle‐like morphology, whereas untreated macrophages displayed a round‐like morphology.^[^ ^43^, ^44^ ^]^ More specifically, SiO_2_@PP‐Cur treatment resulted in the shrinkage and disappearance of tentacles and a round‐like morphology of macrophages. Moreover, LPS stimulation significantly up‐regulated the protein expression level of canonical biomarkers of M1 macrophages (inducible nitric oxide, inducible nitric oxide synthetase (iNOS), and cluster of differentiation 86 (CD86)), which were reversed by SiO_2_@PP‐Cur (Figure 4B). Concurrently, the protein expression level of cluster of differentiation 206 (CD206) and arginine‐1 (Arg‐1), which are canonical biomarkers of M2 macrophages, was significantly elevated after treatment with SiO_2_@PP‐Cur. These observations were in line with the results of immunofluorescent staining (Figure 4C) and flow cytometry (Figure 4D). Collectively, these results suggested that SiO_2_@PP‐Cur exerted a re‐educating effect on macrophages by suppressing M1 polarization but promoting M2 polarization.

Under pathological conditions, aberrant pro‐inflammation is frequently accompanied by permanent oxidative stress, which drives inflammatory cells infiltration, predominately M1 macrophages.^[^ ^22^, ^42^ ^]^ The activation of heme oxygenase 1 (HO‐1) and NAD(P)H: quinine oxidoreductase 1 (NQO‐1) and their upstream regulator, nuclear factor erythroid 2‐related factor 2 (Nrf2), is crucial to inhibit the oxidative stress and inflammatory response.^[^ ^45^ ^]^ As portrayed in Figure 4E, treatment with SiO_2_@PP‐Cur significantly increased Nrf2, HO‐1, and NQO‐1 protein content in a dose‐dependent way, suggesting the participation of Nrf2 signaling in the anti‐inflammatory and anti‐oxidative effect of SiO_2_@PP‐Cur in OA. To summarize, SiO_2_@PP‐Cur significantly inhibited oxidative stress and decreased ROS levels, accompanied by the activation of anti‐inflammatory signaling pathways such as Nrf2. The protein expression of M2 macrophage markers (CD206 and Arg‐1) was elevated, thus driving the formation of an anti‐inflammatory microenvironment. Notably, M2 macrophages can synthesize anti‐inflammatory cytokines in order to remodel the impaired microenvironment to a progenerative microenvironment.

### 2.5 Evaluation of Antiarthritic Efficacy In Vivo

**Figure 5:**
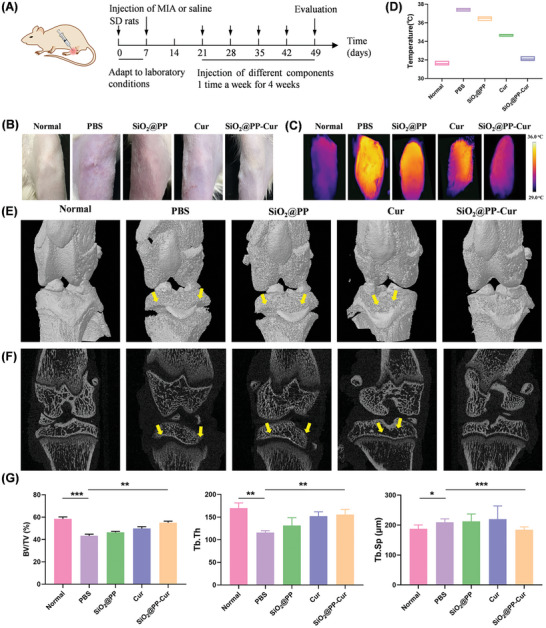
Morphological analysis of knee joints of OA rats after treatment with SiO_2_@PP‐Cur. A) Schematic diagram of the protocol for the construction and treatment of rat OA models. B) Representative digital images and C) thermographic images of joint knees at week 7. D) Quantification of the temperature of the knee joint in the various groups (*n* = 3). E,F) Representative CT scans of healthy and arthritic knees at week 7 (*n* = 3). G) BV/TV quantitative analysis, Tb.Th and Th.Sp of knee joints. Data are expressed as means ± SD. (*n* = 3), p* < 0.05, p**< 0.01, p***< 0.005.

Given the promising anti‐oxidative and anti‐apoptotic impact of SiO_2_@PP‐Cur in vitro, its therapeutic efficacy was assayed in a rat OA model. And before performing the in vivo experiments, we first evaluated the biocompatibility of SiO_2_@PP‐Cur. Via the standard hemolysis assay, it was shown that SiO_2_@PP‐Cur exposure (50 µg·mL^−1^, 1 h) would not cause any obvious hemolysis (Figure S14, Supporting Information), indicating their excellent hemocompatibility. Meanwhile, there was no obvious difference in blood routine parameters (MPV, RDW‐CV, MCHC, MCH, MCV, HCT, HGB, RBC, and WBC) in the SiO_2_@PP‐Cur (100 mg·kg^−1^) groups (Figure S15, Supporting Information). The OA model of rats was established via intra‐articular injection of mono‐iodoacetic acid (MIA) that was demonstrated to achieve pathological progression similar to human OA.^[^ ^46^, ^47^ ^]^ On day 21, the rats were randomly separated into four groups and then received different treatments (PBS, SiO_2_@PP, Cur, and SiO_2_@PP‐Cur) via intra‐articular injection every 7 days for 28 days (**Figure** **5**A). Hyperthermia in knee joints is often associated with OA‐related inflammation due to synovitis and increased subchondral bone activity.^[^ ^48^, ^49^ ^]^ To evaluate the therapeutic efficacy of SiO_2_@PP‐Cur, the degree of swelling and hyperthermia in the knee joints were assessed at the endpoint. As anticipated, severe swelling and hyperthermia were detected in the joints of the knee in OA rats compared with those in healthy rats, indicating that the OA model was successfully established (Figure 5B,C). Compared to the PBS‐treated OA rats, treatment with either SiO_2_@PP or Cur partially relieved swelling and hyperthermia, whereas SiO_2_@PP‐Cur treatment significantly ameliorated these arthritis‐related symptoms. Likewise, the results of micro‐computed tomography (micro‐CT) for the knee joints corroborated the positive therapeutic effects of SiO_2_@PP‐Cur in OA. As portrayed in Figure 5E,F, injection of SiO_2_@PP and Cur marginally attenuated severe bone erosion in the joints. In contrast, SiO_2_@PP‐Cur‐treated rats exhibited a clear bone boundary with a lower degree of bone erosion, particularly compared to bone erosions with irregular surfaces in PBS‐treated rats. The quantitative analysis of Bone Volume/Total Volume (BV/TV), Trabecular Thickness (Tb.Th), and Trabecular Separation (Tb.Sp) were consistent with the alterations observed on CT images (Figure 5G). Lastly, joint aggravation and injury were least severe in the SiO_2_@PP‐Cur group due to the hydration lubricating property of SiO_2_@PP‐Cur and the effective release of curcumin in the inflammatory microenvironment.

### 2.6 In Vivo Regeneration of Damaged Cartilage by SiO_2_@PP‐Cur

**Figure 6:**
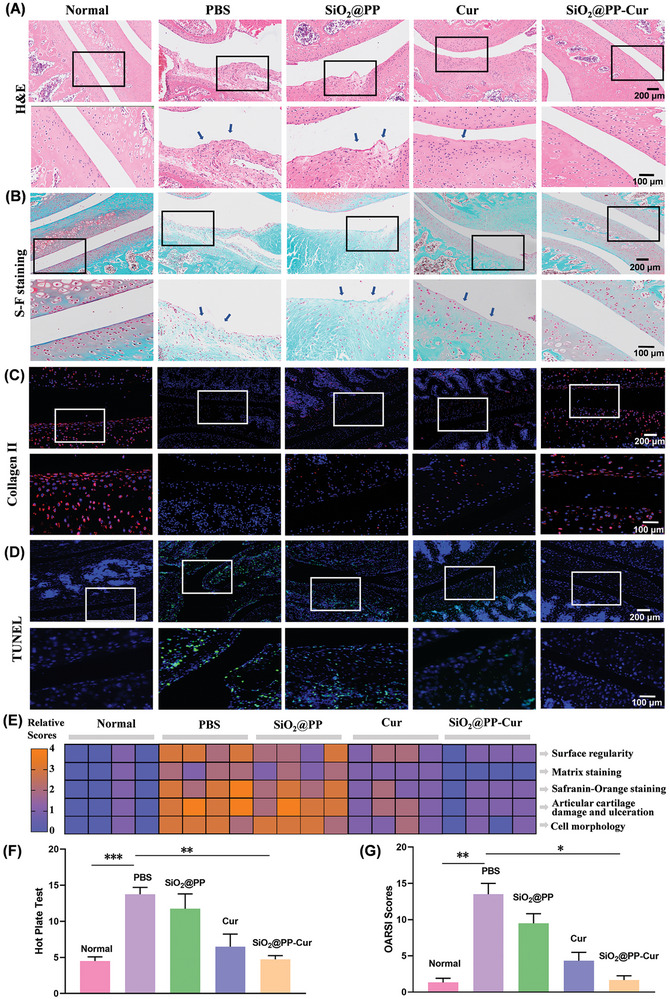
Histological analysis of OA knee joints of rats following supplementation with SiO_2_@PP‐Cur. A) Representative H&E staining and B) Safranin O‐fast green staining of the cartilage sections of osteoarthritic rats following different treatments. C) Representative sections of immunohistochemical staining for collagen II. D) Representative sections of TUNEL staining for apoptotic cells. E) Heatmap of variables of histological scores. F) Hot plate test of rats at 7 weeks. G) OARSI grades of rat joints at 7 weeks. Data are presented as means ± SD. (*n* = 3), p* < 0.05, p**< 0.01, p***< 0.005.

Histological examinations and immunofluorescence analyses were further performed to characterize the therapeutic efficacy of SiO_2_@PP‐Cur. Hematoxylin and eosin (H&E) and Safranin O‐fast green staining exposed the typical symptoms of OA in PBS‐treated OA rats, including severe articular cartilage destruction, inflammatory cell infiltration, and weak distribution of proteoglycans in the extracellular matrix (**Figure** **6**A,B). SiO_2_@PP‐Cur administration rescued the damaged cartilage, as demonstrated by the integrity and smooth cartilage zones in addition to the even distribution of proteoglycans in the extracellular matrix. Moreover, the expression of type II collagen, a major cartilage biomarker, was recovered to the greatest extent in SiO_2_@PP‐Cur‐treated rats compared to other groups (Figure 6C). The decrease in the apoptotic rate of chondrocytes (green staining) was more pronounced in rats treated with SiO_2_@PP‐Cur relative to those treated with SiO_2_@PP and Cur (Figure 6D). Histological scores based on cell morphology, matrix staining, surface regularity, articular cartilage damage, and ulceration, as well as safranin‐orange staining, were employed to assess the treatment efficacy of SiO_2_@PP‐Cur in OA rats (Figure 6E,F). At the same time, the inflammatory OA grading standard (OARSI) was used to score the sections of the knee joint (Figure 6G). The analysis revealed that the scores were lowest in the SiO_2_@PP‐Cur‐treated rats.

**Figure 7:**
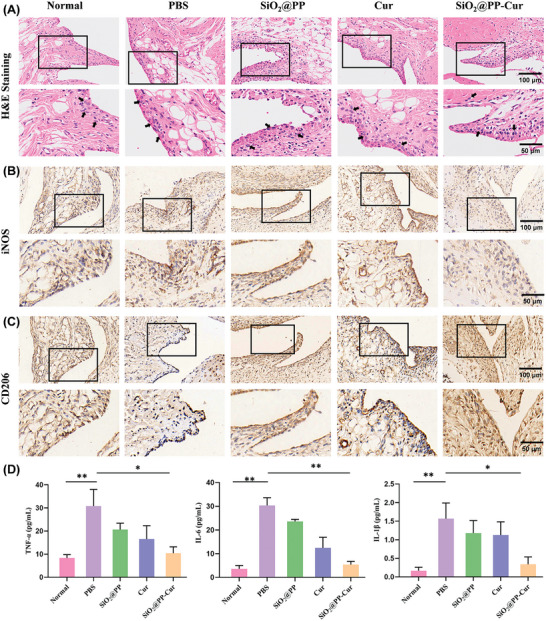
A) H&E staining of synovial tissue. Synovial macrophages stained with B) iNOS and C) CD206 markers. D) Serum levels of TNF‐α, IL‐6, and IL‐1β in the different groups. Data are presented as means ± SD. (*n* = 3), p* < 0.05, p**< 0.01, p***< 0.005.

The overactivation of defective M1 macrophages is conducive to the formation of a pro‐inflammatory microenvironment.^[^ ^23^, ^25^ ^]^ Therefore, immunohistochemical staining of iNOS (a canonical biomarker of M1 macrophages) and CD206 (a canonical biomarker of M2 macrophages) was carried out on rat knee joints following the relevant treatment. As depicted in **Figure** **7**A–C, in the SiO_2_@PP‐Cur­treatment group, the proportion of iNOS‐positive macrophages dramatically declined, whereas the percentage of CD206‐positive macrophages was relatively higher. In the synovial tissue sections, dual immunofluorescence staining was performed using F4/80/CD86 for M1 macrophages and F4/80/CD206 for M2 macrophages (Figure S17, Supporting Information). In the SiO_2_@PP‐Cur group, there was a notable decrease in the distribution of CD86 positive macrophages, whereas the distribution of CD206 positive macrophages was significantly enhanced. Taken together, the SiO_2_@PP‐Cur group effectively reversed the adverse pro‐inflammatory condition and reprogrammed the macrophages toward a pro‐regenerative M2 phenotype. Moreover, the levels of three canonical pro‐inflammatory cytokines, TNF‐α, IL‐1β, and IL‐6, were significantly decreased in SiO_2_@PP‐Cur‐treated rats, comparable to the corresponding values in healthy rats (Figure 7C). Notably, the therapeutic effect of the SiO_2_@PP‐Cur group was superior given that it possesses hydration‐lubricating properties, is coated with a ROS‐scavenging polydopamine layer, and can secrete curcumin to induce anti‐inflammatory effects, particularly in an inflammatory microenvironment. Therefore, intra‐articular injection of SiO_2_@PP‐Cur can effectively lubricate joints and delay osteoarthritis development and progression. Clinically, curcumin intra‐articular injection had a rather modest impact on osteoarthritis relief. Mechanically, curcumin spontaneously reacts with synovial components, leading to an impaired antioxidative capacity. This curcumin deficiency was effectively resolved by delivering curcumin via SiO_2_@PP‐Cur. More importantly, treatment with SiO_2_@PP‐Cur did not induce any observable tissue damage, indicating its satisfactory biocompatibility (Figure S14, Supporting Information). Taken together, SiO_2_@PP‐Cur exhibited an excellent regenerative effect on the damaged OA cartilage by minimizing the apoptosis of chondrocytes via re‐modulating the OA microenvironment.

## 3 Conclusion

Herein, we developed a SiO_2_@PP‐Cur core‐brush nanoplatform and demonstrated its potential in OA therapy via its synergistic anti‐inflammatory and lubricating effects. Furthermore, SiO_2_@PP‐Cur promoted M1 macrophage polarization to the M2 phenotype and increased the concentration of anti‐inflammatory cytokines, thereby remodeling the damaged microenvironment. Intra‐articular injection of SiO_2_@PP‐Cur into MIA rats resulted in higher therapeutic efficacy than SiO_2_@PP or Cur, which was demonstrated by effectively alleviating cartilage wear, reducing the inflammatory reaction, and limiting articular cartilage breakdown and the degradation of extracellular matrix. Overall, these results demonstrate the therapeutic potential of SiO_2_@PP‐Cur as a safe and reliable core‐brush nanoplatform for the systematic treatment of OA.

## 4 Experimental Section

### Materials

2‐methacryloyloxyethyl phosphorylcholine and (3‐Acrylamidophenyl) boronic acid were purchased from Mackllin, China. Tris (2‐dimethylaminoethyl) amine (Me_6_TREN), Curcumin (Cur), dopamine hydrochloride, and copper (I) bromide (CuBr) were procured from J&K Scientific Ltd. Beijing, China. Lipopolysaccharide (LPS) was acquired from Sigma‐Aldrich (St. Louis, USA). Interleukin‐4 (IL‐4) was obtained from MedChemExpress (Monmouth Junction, NJ, USA). Dulbecco's modified eagle medium (DMEM), Dulbecco's modified eagle medium/Ham's F 12 nutrient medium (DF12), fetal bovine serum (FBS), penicillin/streptomycin solution, and trypsin were obtained from Gibco (Grand Island, NY). Hydrogen peroxide analysis kit, cell counting kit‐8 (CCK‐8), 2,7‐dichlorofluorescin diacetate (DCFH‐DA), 4′,6‐diamidino‐2‐phenylindole (DAPI), RIPA buffer, and Annexin V‐FITC/PI apoptosis detection kit were obtained from Beyotime Biotechnology (Shanghai, China). COX‐2 antibody, GAPDH antibody, iNOS antibody, CD86 antibody, CD206 monoclonal antibody, and Arg‐1 antibody were acquired from ABclonal Biotechnology (Woburn, MA). Nrf2 antibody, HO‐1 antibody, NQO1 antibody, and β‐actin antibody were purchased from Abcam (Cambridge, UK). CD11b monoclonal antibody, F4/80 monoclonal antibody, CD206 (MMR) monoclonal antibody, and CD86 monoclonal antibody were obtained from Thermo Fisher Scientific Inc., USA.

### Synthesis of SiO_2_@PP‐Cur

Pristine SiO_2_ was ultrasonically dispersed in a 10 mm Tris solution containing dopamine (1 mg·mL^−1^). Self‐polymerization‐induced dopamine was deposited for 5 h at room temperature. Following this, the prepared SiO_2_@PDA was centrifuged, washed with water and ethanol, then dewashed in an oven at 35 °C. Then, SiO_2_@PDA was added to 100 mL of anhydrous dichloromethane, after which triethylamine (20.4 mmol, 2.83 mL) was added. Next, BiBB (16.9 mmol, 2 mL) was added to 10 mL dichloromethane. After 5 h of stirring, the nanoparticle was alternately washed with ethanol and water and dewashed in an oven at 35 °C to generate SiO_2_@PDA‐Br. Using SI‐ATRP, the immobilized bromide group acted as an initiator to stimulate the synthesis of MPC and PBA brushes, during which CuBr/PMDETA (molar ratio of 1:1) served as a catalyst. MPC monomer (0.25 g, 0.85 mmol), PBA monomer (0.16 g, 0.85 mmol), and PMDETA (16.9 µL, 85 µmol) were dissolved in a mixture containing 7 mL of DMF and water (molar ratio of 3:1) in a Schlenk tube. Afterward, CuBr (12.2 mg, 85 µmol) was supplemented with N_2_, then, polymerization was initiated at 60 °C. After 10 h, polymerization was terminated when it was subjected to the air. The target SiO_2_@PP was centrifuged and freeze‐dried. SiO_2_@PP was dispersed in a mixture of DMF and water (molar ratio of 3:1) at a 10 mg·mL^−1^ concentration, and curcumin was liquified by dissolving it in ethanol to reach a final concentration of 1 mg·mL^−1^. Then, the above two solutions were mixed and gently stirred for 4 h in the dark. Finally, the obtained SiO_2_@PP‐Cur was centrifuged and freeze‐dried.

### Drug Loading Content

The supernatant was collected, diluted with methanol, and measured by High‐Performance Liquid Chromatography (HPLC). The amount of curcumin was calculated by standard curve. The drug loading content (DLC) of curcumin was calculated using the following formula:

(1) $$\begin{array}{ccc} \mathrm{DLC}\left(\%\right) & = & (\mathrm{weight}\;\mathrm{of}\;\mathrm{curcu}\min\;\mathrm{encapsulated}\;\mathrm{inside}\;\mathrm{Si}O_{2}@\mathrm{PP}\\ & & -\;\mathrm{CurNPs}/\mathrm{total}\;\mathrm{weight}\;\mathrm{of}\;\mathrm{Si}O_{2}@\mathrm{PP}-\mathrm{CurNPs})\times 100\% \end{array}$$

### Characterization of SiO_2_@PP‐Cur

Chemical structures of samples were determined with ^1^H NMR (Bruker Avance 400 MHz NMR spectrometer), Fourier transform infrared spectrometry (FT‐IR, Bruker Tensor 27 spectrometer), and X‐ray photoelectron spectroscopy (XPS, Thermo Fisher Scientific Escalab 250 spectrometer). UV–vis adsorption spectra were monitored by a TU‐1901 spectrophotometer. Thermogravimetric analyses (TGA) were tested by a TA SDTQ600 tool from 30 to 800 °C under N_2_. Transmission electronic microscopy (TEM) images were captured via an HT7700 transmission electron microscope (HITACHI, Japan).

### Tribological Test

The tribology experiment was carried out using a complete tester for material surface properties (CFT‐1, Lanzhou Zhongke Kaihua Technology Development Co., Ltd., Lanzhou, China). A polished Ti6Al4 V disk was utilized as the lower specimen, and the polytetrafluoroethylene (PTFE) ball was utilized as the upper sample. All tests were carried out using the reciprocating mode for a period of 30 min.

### In Vitro ROS Scavenging Assays

H_2_O_2_, DPPH·, ABTS+· O_2_^·−^, and ·OH levels were monitored to assess the scavenging capability of ROS of the nano‐formulations. The H_2_O_2_ scavenging activity was evaluated utilizing a hydrogen peroxide analysis kit. Nano‐formulations and DPPH· (50 µm) were mixed in ethanol for 30 min. DPPH· absorption was then assessed at 517 nm. 7 mM ABTS solution was added to 2.45 mm potassium persulfate and left to react overnight to activate ABTS+· radicals. Nano‐formulations with the adjusted working concentrations were added to the ABTS+· radical solution and left to react for 10 min. Lastly, the absorption of ABTS+· radicals were detected at 734 nm. ·OH scavenging activity was explored by using Hydroxyl Free Radical Scavenging Capacity Assay Kit. Briefly, Nano‐formulations and Kit working solution (300 µL) were mixed for 1 h in dark, and the supernatant absorbance at 536 nm was recorded. O_2_^−^ scavenging activity was detected with Total Superoxide Dismutase Assay Kit (WST‐8). Nano‐formulations were mixed with 160 µL WST‐8 working solution, and then 20 µL of xanthine oxidase solution was added and incubated for another 30 min. Later, the absorbance at 450 nm was measured.

### Cell Cytotoxicity Assay

Raw264.7 cells were cultivated in 5% CO_2_ at 37 °C in a hyperglycemic Dulbecco's modified eagle's medium (DMEM, Wisent, China) supplemented with 10% inactivated FBS and 1% penicillin/streptomycin. Macrophages were inoculated with SiO_2_@PP‐Cur in 96‐well plates at a density of 10 000 cells/well for 24 h. Then, 10 µL of CCK‐8 solution was added to each well of the plate and the cells were cultured for another 2 h. The absorbance of the solution was measured at 450 nm using a microplate reader (Varioskan LUX, Thermo fisher scientific, USA).

### Live/Dead Staining Assay

Cell viability was determined using live/dead staining (Beyotime, China). Live cells were stained with calcein AM, while dead cells were stained with propidium iodide (PI). RAW264.7 cells were seeded in 24‐well plates at a density of 100 000 cells per well and treated with SiO_2_@PP‐ for 24 hours at 37 °C in a 5% CO_2_ atmosphere. The medium was aspirated and the cells were thoroughly washed with PBS. Staining dyes were then added to each well. After incubating for 30 min at room temperature, the cells were examined using fluorescence microscopy (Axiovert, Zeiss, Germany).

*Western Blotting Analysis*: RAW264.7 cells were cultured in six‐well plates for 12 h before being treated with LPS (1 µg·mL^−1^) and various nano‐formulations at differing concentrations (SiO_2_@PP‐Cur: 50 µg·mL^−1^ and equivalent doses of other nano‐formulations) for 24 h. The cells were washed three times with PBS and lysed by adding 200 µL of RIPA buffer containing 1% protease inhibitor mixture on ice for 30 min. Proteins were extracted by centrifugation at 12 000 g for 15 min, and concentrations were quantified using a BCA protein assay kit (Thermo Fisher, USA). Samples containing 30 µg of protein were processed through sodium dodecyl sulfate‐polyacrylamide gel electrophoresis (SDS‐PAGE). Then the PVDF membranes were blocked using 5% skimmed milk, incubated overnight with primary antibodies at 4 °C, washed thrice with TBST for 10 minutes, and treated with diluted secondary antibodies for 1 h. Protein bands were visualized by enhanced chemiluminescence and captured using a ChemiDoc ImageLab system (Bio‐Rad, USA).

### In Vitro Cell Apoptosis Analysis

RAW264.7 cells were seeded at 400000 cells per well in six‐well plates and cultured for 12 h. Following initial culture, the cells were treated with 1 µg mL^−1^ LPS and then exposed to various nano‐formulations at different concentrations for 12 h. Cells were harvested by centrifugation at 2000 rpm for 5 min and resuspended in 500 µL of 1× binding buffer. For apoptosis analysis, the cells were stained with membrane‐bound protein V‐FITC and PI staining solution, incubated at room temperature for 15 minutes in the dark, and subsequently analyzed by flow cytometry.

### In Vitro Evaluation of Macrophage Polarization

RAW264.7 macrophages were stimulated with LPS for 24 h to display the M1 phenotype. At the same time, M1 macrophages were additionally treated with nano‐formulations at various concentrations (SiO_2_@PP‐Cur: 50 µg·mL^−1^ and equivalent doses of other nano‐formulations) for 24 h. Afterward, cell polarization was detected by western blot, flow cytometry, and immunofluorescence assay. For western blot assay, the expression of M1 marker (iNOS and CD86) and M2 marker (CD206 and Arg‐1) proteins were detected.

For immunofluorescence assay, macrophages were fixed in 4% paraformaldehyde at room temperature for 15 min, followed by PBS washed. Cells were then permeabilized with 0.25% Triton X‐100 on ice for 10 min. After blocking with bovine serum albumin for 30 min, cells were incubated overnight at 4 °C with primary antibodies: anti‐iNOS (Abclonal, China) and anti‐CD206 (Proteintech, China). Then, the cells were washed with PBS and incubated with a fluorescence‐conjugated secondary antibody (Abcam, USA) for 1 h at room temperature. After this, DAPI (Beyotime, China) was used to label the nuclei after washing with PBS. Fluorescence images were acquired using a confocal laser scanning microscope (Celldiscoverer 7, Zeiss, Germany).

For flow cytometry analysis, CD86 was selected to mark the M1 phenotype and CD206 for the M2 phenotype. Macrophages were first gated by F4/80 positive cells, and then CD86 and CD206 expression levels were analyzed in cells treated with different treatments.

### Animal Model

SD rats (male, 6–8 weeks) were acquired from Vital River Laboratories (Beijing, China). The study was approved by the Institutional Animal Care and Use Committee of the Shandong Medicinal Biotechnology Center. Briefly, 5% mono‐iodoacetic acid (MIA) was mixed and intraarticularly injected into the knees of rats to establish the OA model. Two weeks after following the construction of the OA model, the rats were randomly assigned to five groups for treatment: 1) Normal+ PBS, 2) MIA+PBS, 3) MIA+SiO_2_@PP, 4) MIA+Cur, and 5) MIA+SiO_2_@PP‐Cur (*n* = 3 per group). All treatments were administered by intra‐articular injection. 100 mg·kg^−1^ of SiO_2_@PP‐Cur was injected in rats of the treatment group once weekly. Rats were administered the treatment every seven days and were euthanized on day 28. Finally, the temperature of the knee joint was recorded using a digital infrared thermal imaging camera prior to euthanasia.

### ELISA Assay

Concentrations of TNF‐𝛼, IL‐6, and IL‐1β in the serum of SD rats were determined with ELISA assay. At the end of the efficacy experiments, blood from rat was collected and left to stand for 4 h, followed by centrifugation at 3000 rpm for 10 min to obtain 300 µL serum. Finally, levels of TNF‐𝛼, IL‐6, and IL‐1β were determined according to the steps in ELISA kit instructions (Boster, China).

*Micro‐CT Imaging*: The knee joints of rats were first collected and immediately fixed in 4% paraformaldehyde. They were subsequently scanned with a high‐resolution micro‐CT scanner (Skyscan 1172, Bruker, Belgium). The bone volume/total volume (BV/TV) value, the bone surface mineral density (BS/TV), and the trabecula number (Tb. N) were analyzed by using Mimics software.

### Histological Staining Analysis

For histological analysis, at predetermined time points, knee joints from the hind legs of euthanized rats were harvested and fixed in 4% paraformaldehyde for 48 h. The samples were then decalcified in 10% EDTA solution for 1 month, embedded in paraffin, and sectioned. Tissue sections underwent H&E and Safranin O‐fast green staining for histopathological assessment. Cartilage damage and synovial inflammation were evaluated semi‐quantitatively by three blinded observers.

For immunofluorescence assay, sections were incubated overnight at 4 °C with primary antibodies against CD206, CD86, or Collagen II (Servicebio, China). This was followed with a donkey anti‐rat Alexa 647 secondary antibody (Servicebio, China) for 2 h at room temperature incubation. Macrophage polarization at the joint site was assessed using an F4/80 label. Sections were further processed with a TUNEL apoptosis detection kit (Servicebio, China) and nuclei were counterstained with DAPI. Stained sections were examined using a slide scanning system (Slideview vs200, Olympus, Japan).

For immunohistochemistry assay, sections were deparaffinized and subjected to antigen retrieval using antigen repair buffer. Blocking was performed with 3% hydrogen peroxide and 3% BSA. Sections were then incubated with primary antibodies against iNOS and CD206 (Servicebio, China) overnight at 4 °C. This was followed by incubation with biotinylated secondary antibody for 1 h and color development by incubation with DAB, and then the reaction was stopped in distilled water and stained with hematoxylin.

To evaluate the systemic toxicity of nanoparticles in rats, tissues of major organs such as heart, lungs, liver, kidney, and spleen were collected and fixed in 4% paraformaldehyde for 48 hours for H&E staining and pathological analysis. The blood of rats was also collected for the detection and analysis of routine blood biochemical indices.

### Statistical Analysis

All values were presented as mean ± SD (standard deviation). All statistical analysis was performed utilizing Student's *t*‐test. p* < 0.05, p** < 0.01, p*** < 0.005, and p**** < 0.001 were considered statistically significant.

## Conflict of Interest

The authors declare no conflict of interest.

## Supporting information



Supporting Information

## Data Availability

The data that support the findings of this study are available in the supplementary material of this article.
