# Supplementary material for: A Core‐Brush Nanoplatform with Enhanced Lubrication and Anti‐Inflammatory Properties for Osteoarthritis Treatment
Source: Adv Sci (Weinh). 2024 Nov 1;11(47):2406027. doi: 10.1002/advs.202406027 (PMC11653621; doi:10.1002/advs.202406027)
Supplement: Supplementary file 1 — Supporting Information [file ADVS-11-2406027-s001.docx]

Supporting Information

**A core-brush nanoplatform with enhanced lubrication and anti-inflammatory properties for** **osteoarthritis treatment**

Yingying Liu,^#^ Zhiyan Ma,^#^ Xin Wang, Jiaming Liang, Linlin Zhao, Yingyu Zhang, Jiayu Ren, Shuping Zhang,* Yajun Liu*


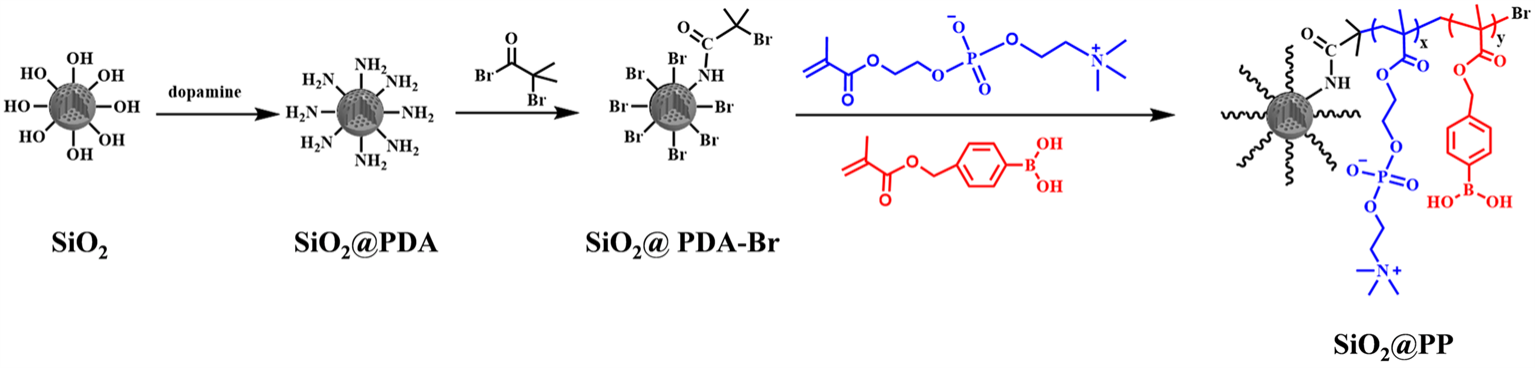


**Figure S1.** The fabrication process of SiO_2_@PP.


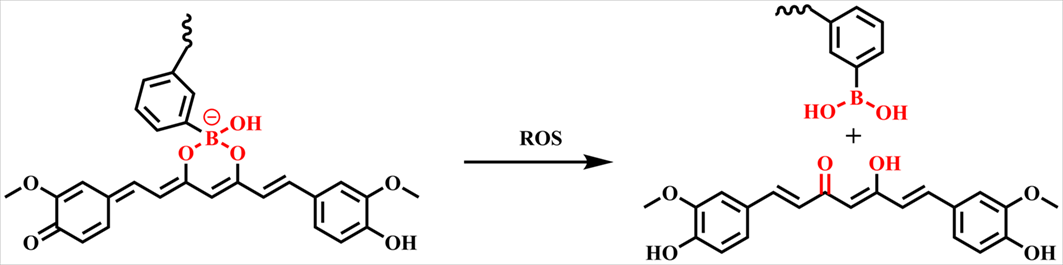


**Figure S2**. The controllable drug release of SiO_2_@PP-Cur.





**Figure S3**. The TGA spectra of SiO_2_, SiO_2_@PDA and SiO_2_@PP.


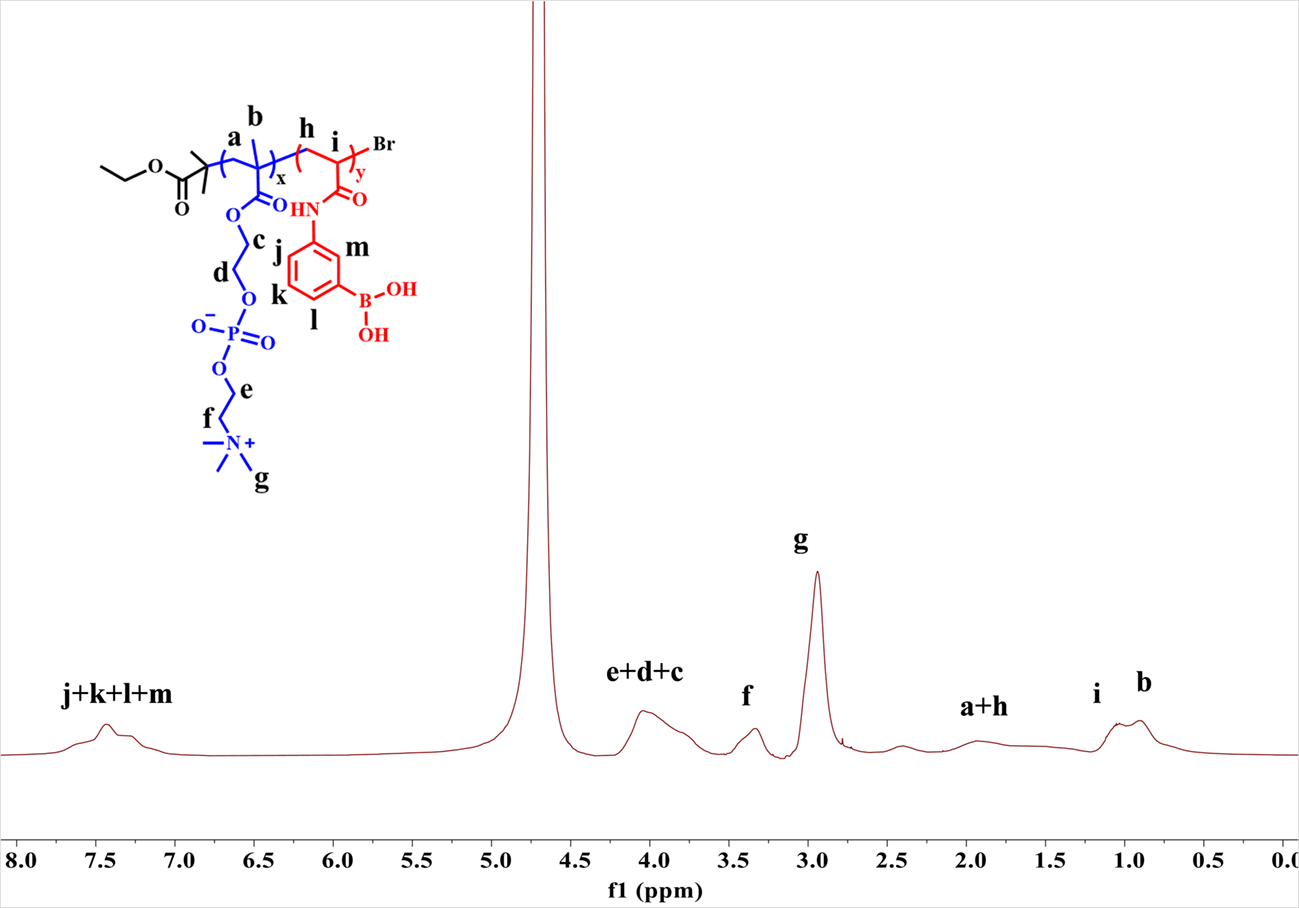


**Figure S4.** ^1^H NMR spectrum of PMPC-PBA co-polymer brush.


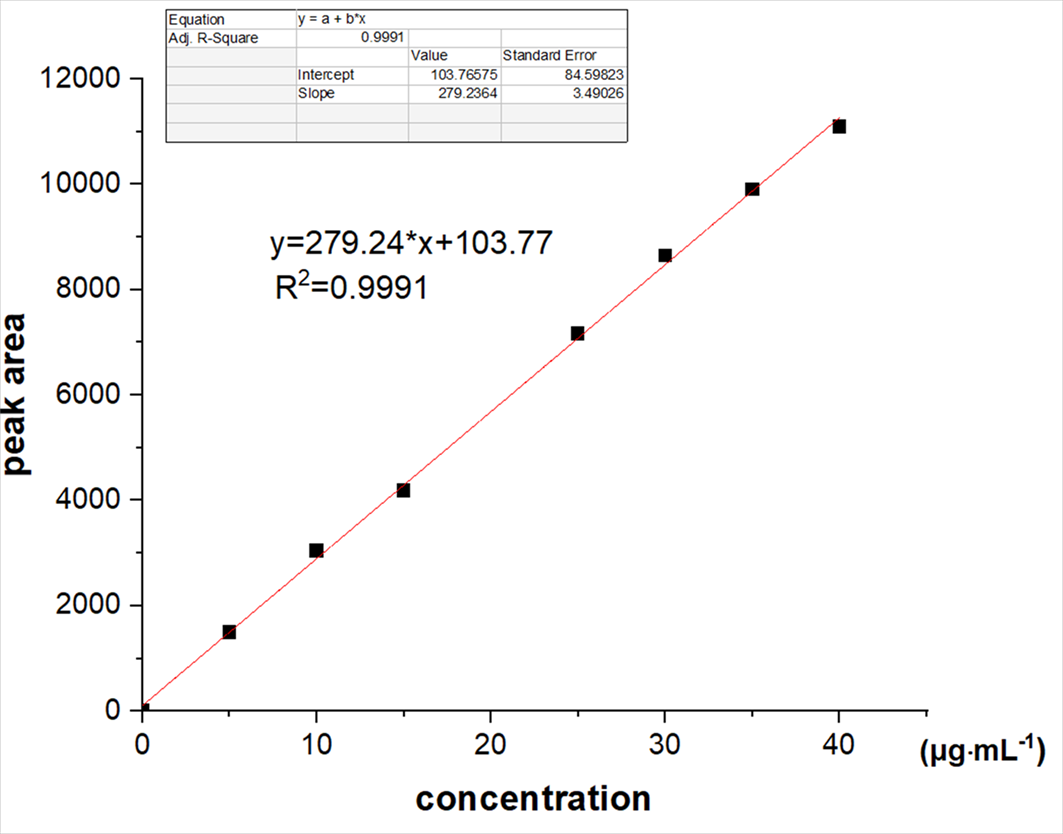


**Figure S5.** Standard curve of curcumin.


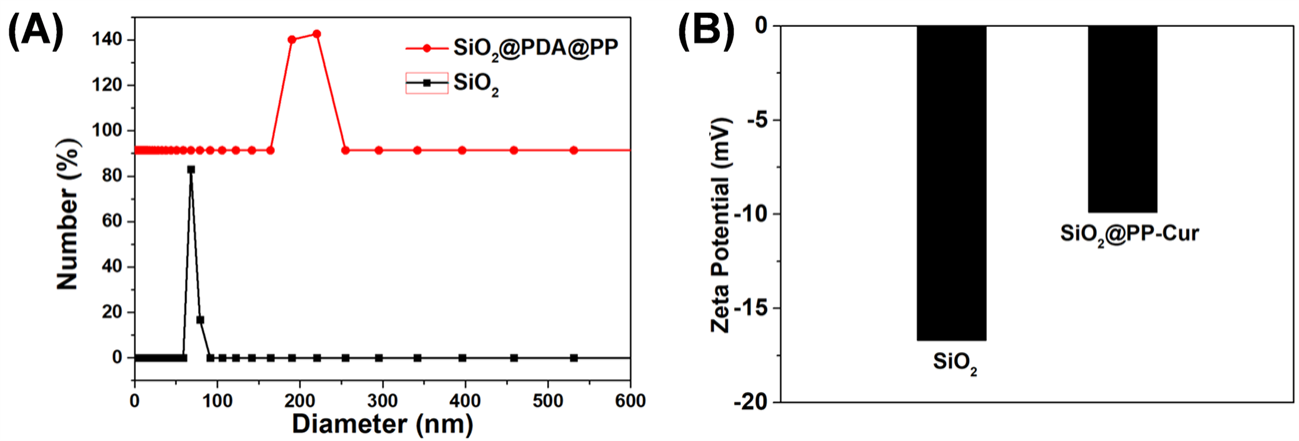


**Figure S6.** Hydrodynamic diameters a) and the zeta-potential b) of SiO_2_ and SiO_2_@PP-Cur after 14-day incubation with PBS.


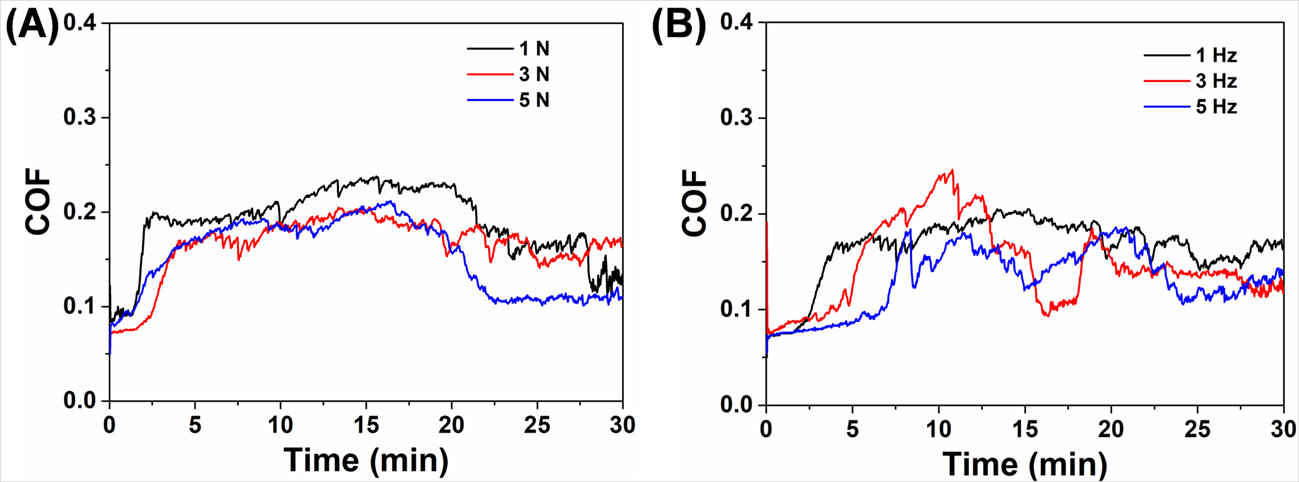


**Figure S7.** The COF-time plots of SiO_2_@PP-Cur under (A) different loads (5 mg·mL^-1^, 3 Hz), (B) different reciprocating frequencies (5 mg·mL^-1^, 5 N).


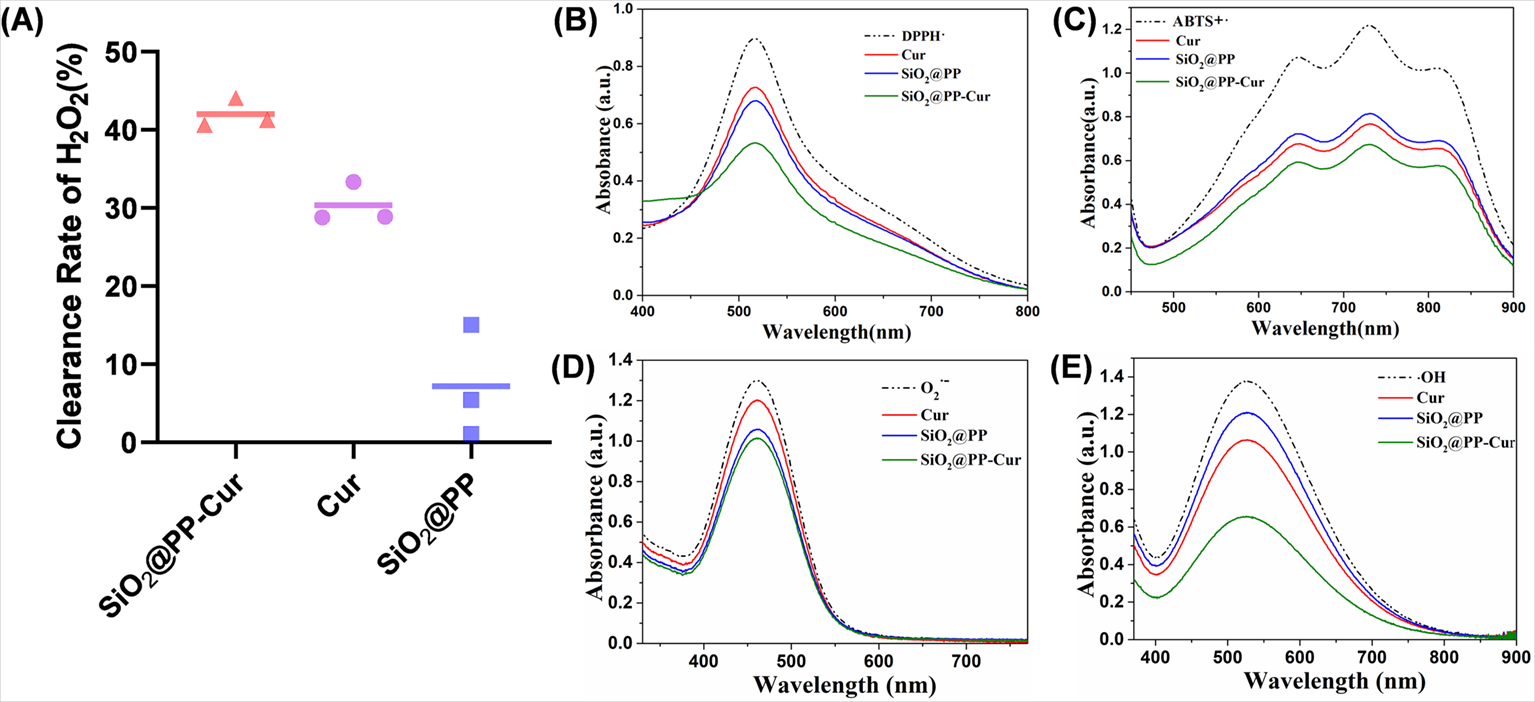


**Figure S8.** (A) H_2_O_2_ scavenging effect of various nano-formulations at the same conditions after storage for 6 months. (B) DPPH· radical (C) ABTS+· radical (D) O_2_^·-^ radical and (E) ·OH radical scavenging effect of nano-formulations after storage for 6 months.


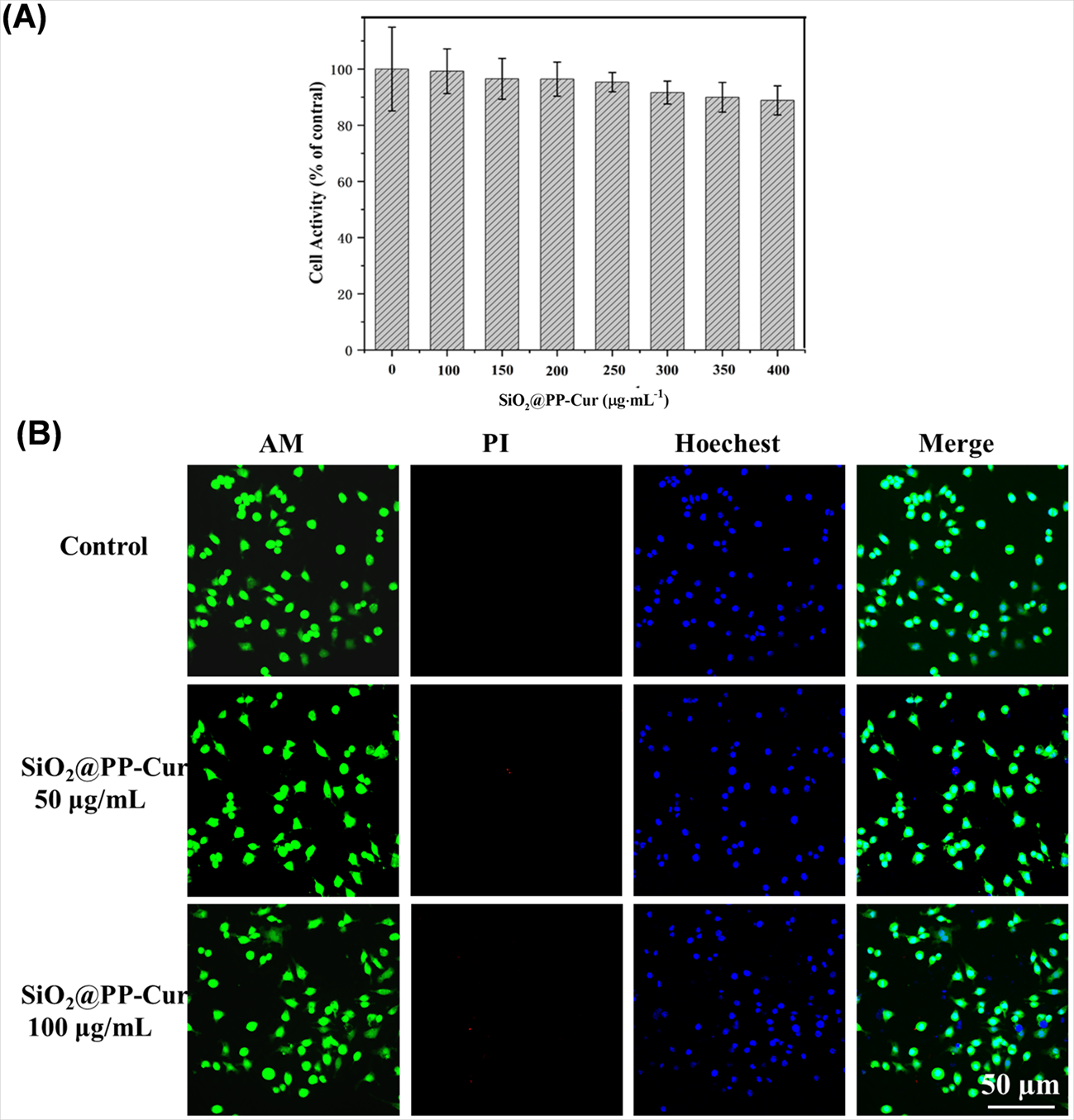


**Figure S9**. (A) Cytotoxicity SiO_2_@PP-Cur on macrophages examined with Cell Counting Kit-8. (B) Live/Dead staining (AM/PI) of macrophages co-cultured with SiO_2_@PP-Cur.


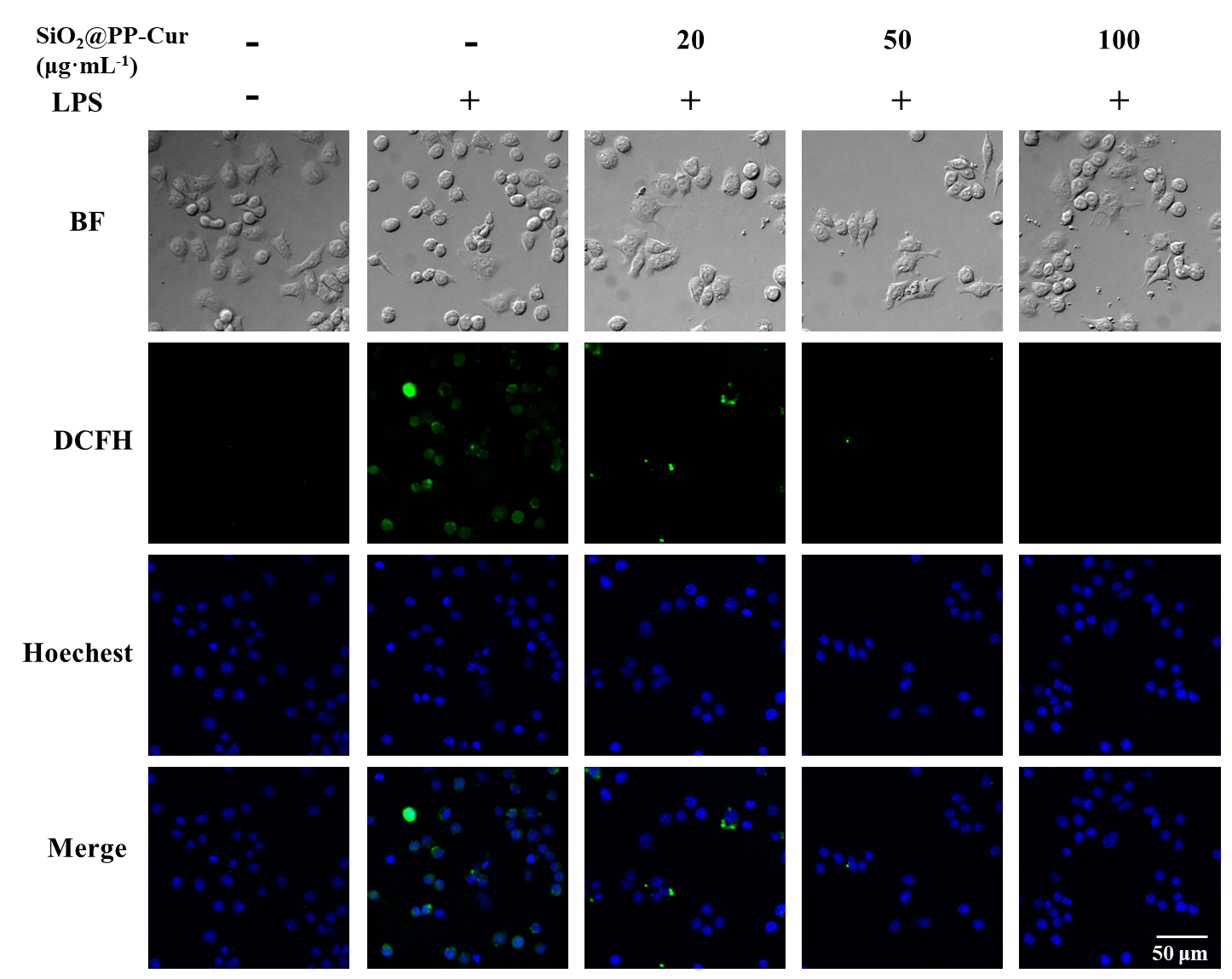


**Figure S10.** ROS levels in macrophages after treatment with different concentrations of SiO_2_@PP-Cur visualized by CLSM (concentration of SiO_2_@PP-Cur: μg·mL^-1^).


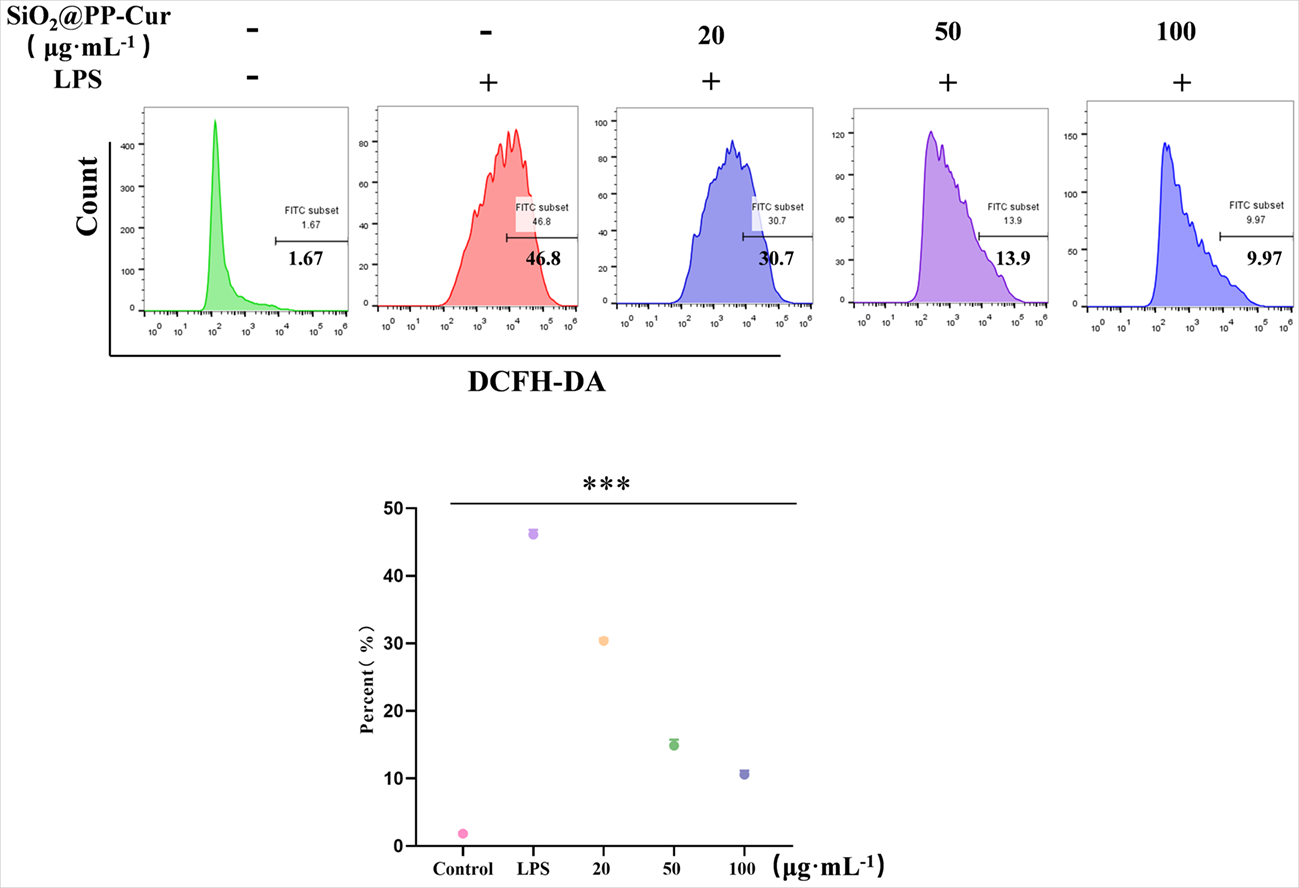


**Figure S11.** ROS levels in macrophages analyzed by flow cytometry.


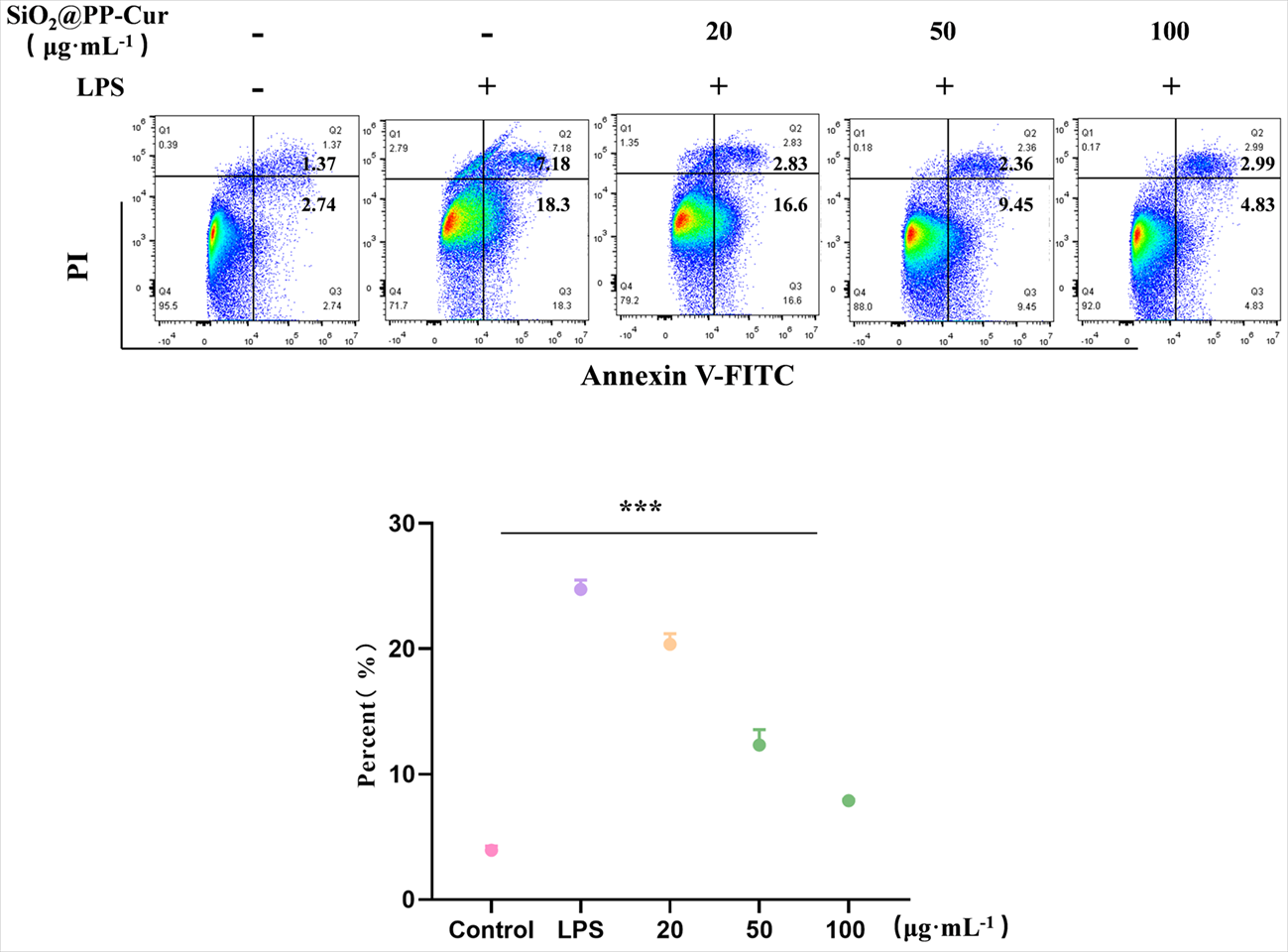


**Figure S12.** Flow cytometry analysis of the number of apoptotic cells.


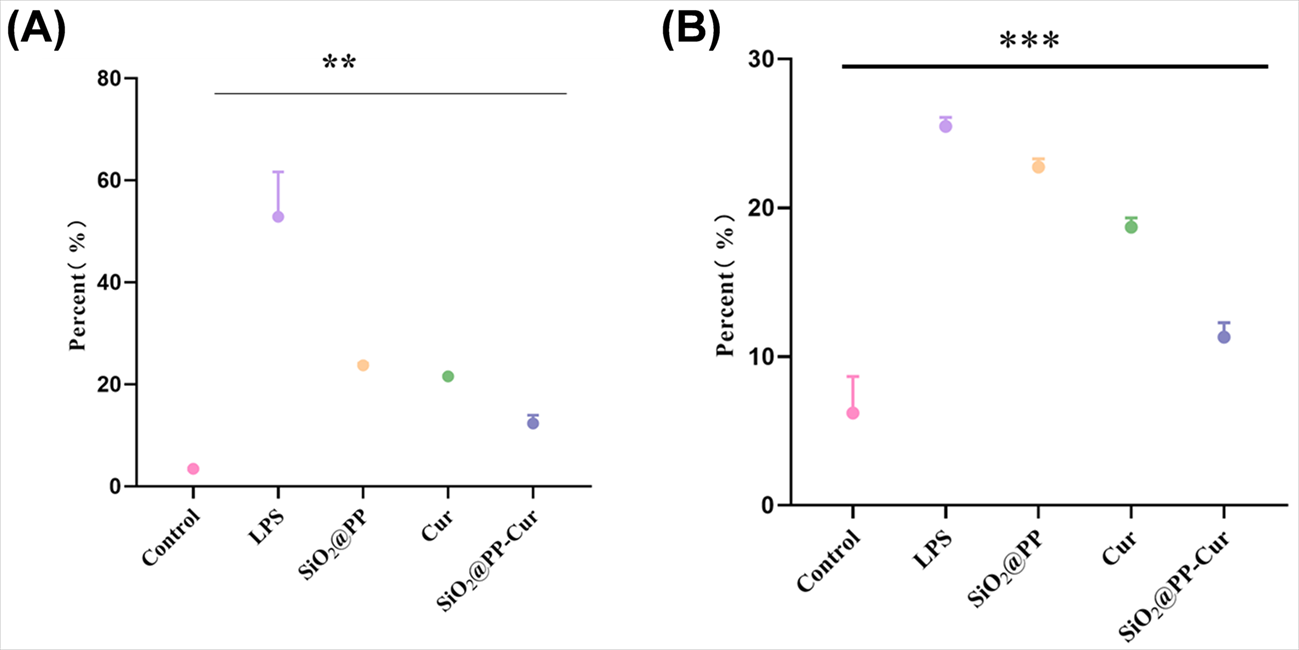


**Figure S13.** (A) Quantitative analysis of ROS levels in Raw 264.7 cells analyzed by flow cytometry. (B) Quantitative analysis of the number of apoptotic cells in Raw 264.7 cells analyzed by flow cytometry.


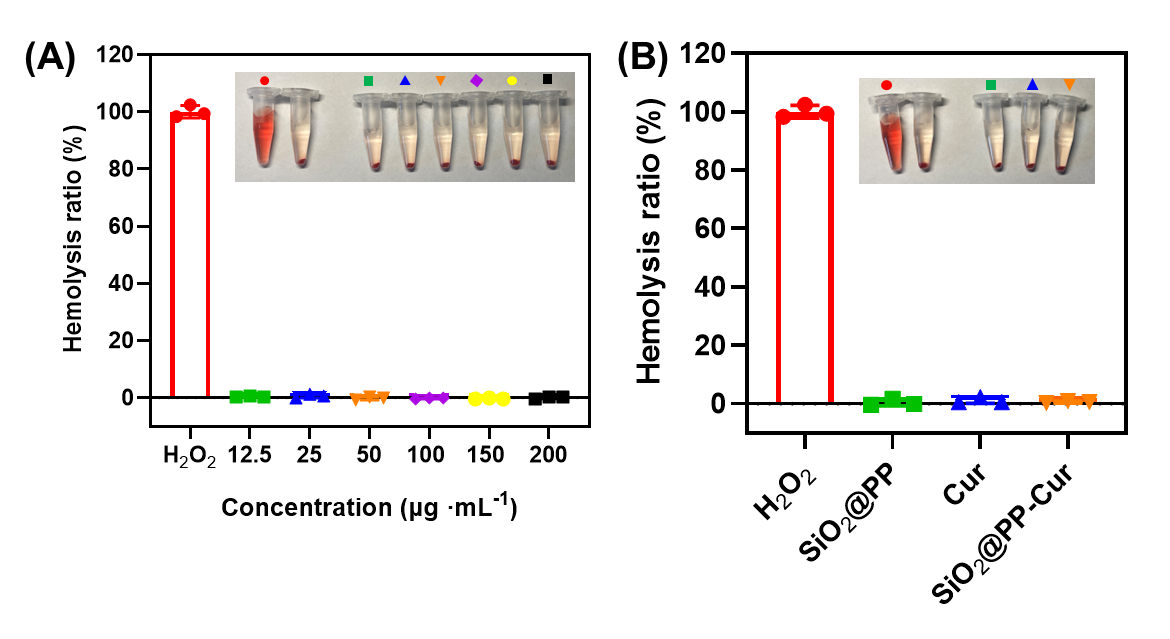


**Figure S14.** Hemolysis ratios of mouse blood after incubation with (A) SiO2@PP-Cur (B) various materials at 37 oC for 1 h. The blood incubated with distilled water was set as 100% hemolysis.

**Figure S15.** Hematological parameters of various rat groups were treated with SiO_2_@PP-Cur (100 mg·kg^-1^) for different days. MPV: mean platelet volume, RDW-CV: red blood cell distribution width, MCHC: mean corpuscular/cellular hemoglobin concentration, MCH: mean corpuscular hemoglobin, MCV: mean red cell volume, HCT: hematocrit, HGB: hemoglobin concentration, RBC: red blood cell, and WBC: white blood cell.


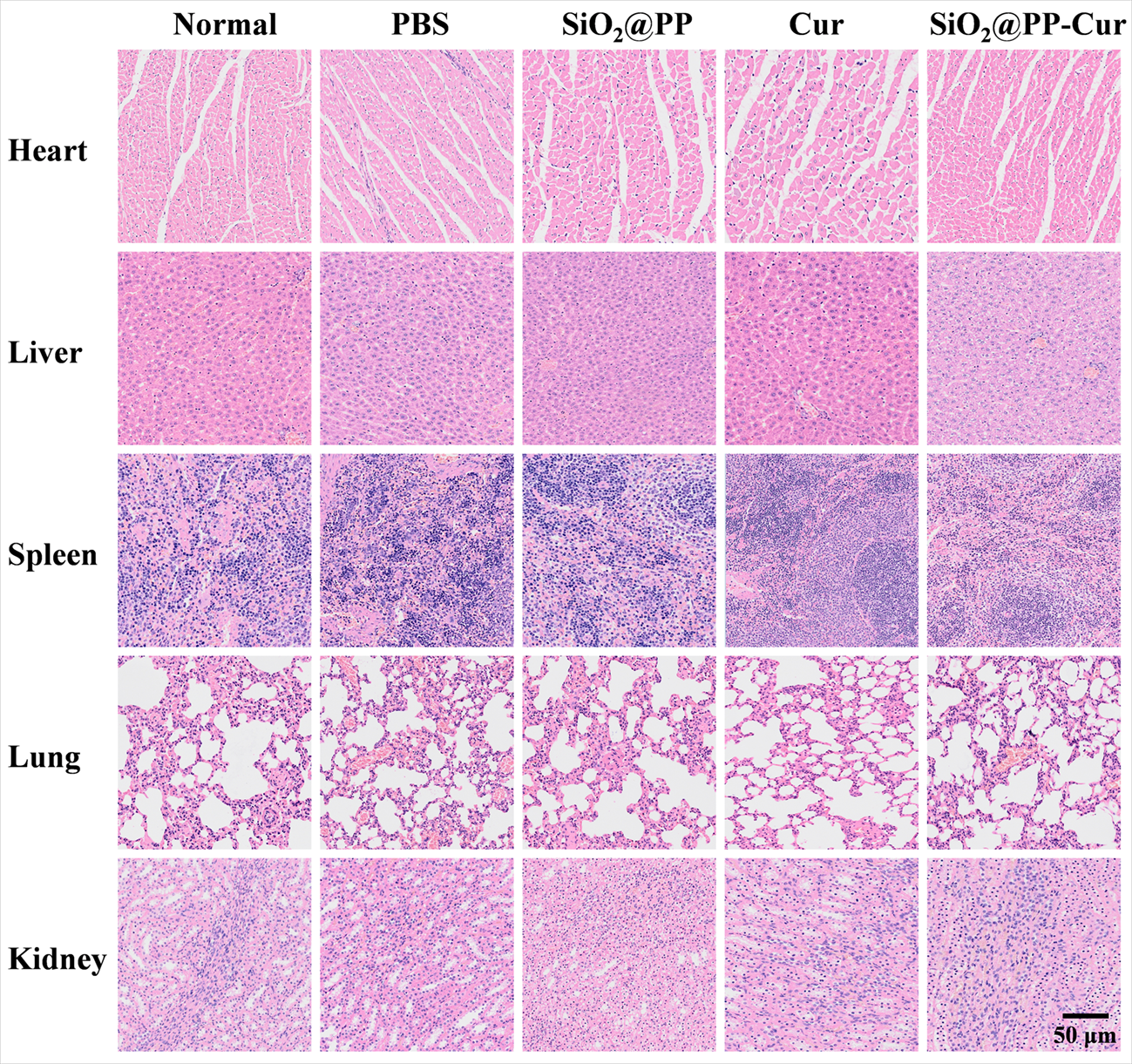


**Figure S16.** H&E staining of main tissues in different treatment groups (scale bar: 50 µm).


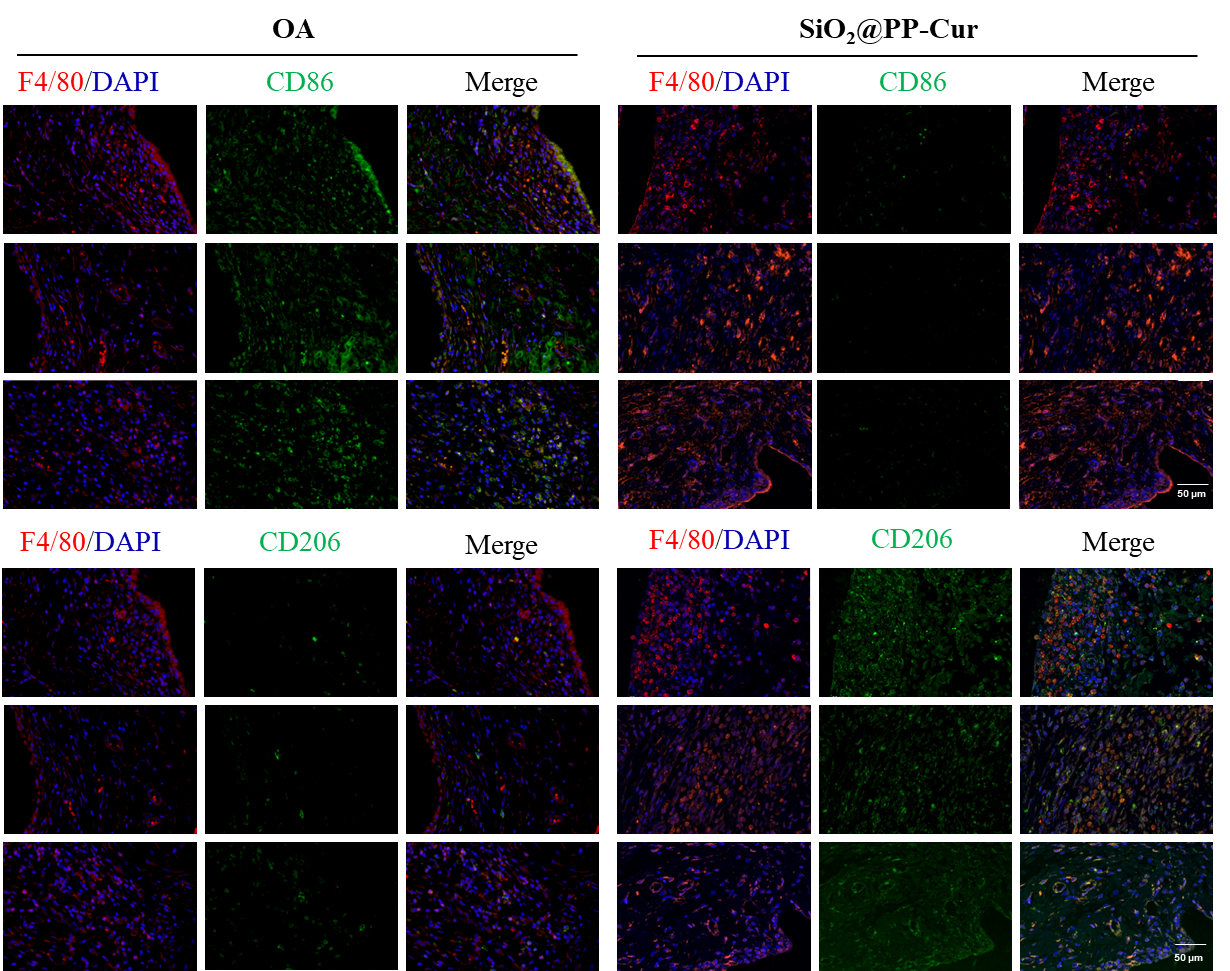


**Figure S17.** Images of immunofluorescent staining of F4/80 (macrophage marker), CD86 (M1 marker), and CD206 (M2 marker) of synovial sections (scale bar: 50 µm).
